# Unraveling the metabolic potential and roles of reductases in the omicsynin biosynthetic gene cluster

**DOI:** 10.1007/s13659-025-00560-5

**Published:** 2026-01-09

**Authors:** Yihong Li, Jie Fu, Hongmin Sun, Yu Du, Shuyi Si, Yuhuan Li, Xingxing Li, Jiandong Jiang, Bin Hong

**Affiliations:** 1 CAMS Key Laboratory of Synthetic Biology for Drug Innovation, Institute of Medicinal Biotechnology, Chinese Academy of Medical Sciences & Peking Union Medical College, No.1 Tiantan Xili, Beijing, 100050 China; 2 NHC Key Laboratory of Biotechnology for Microbial Drugs, Institute of Medicinal Biotechnology, Chinese Academy of Medical Sciences & Peking Union Medical College, No.1 Tiantan Xili, Beijing, 100050 China; 3 CAMS Key Laboratory of Antiviral Drug Research, Institute of Medicinal Biotechnology, Chinese Academy of Medical Sciences & Peking Union Medical College, No.1 Tiantan Xili, Beijing, 100050 China; 4 State Key Laboratory of Bioactive Substances and Functions of Natural Medicines, Institute of Medicinal Biotechnology, Chinese Academy of Medical Sciences & Peking Union Medical College, No.1 Tiantan Xili, Beijing, 100050 China

**Keywords:** Omicsynins, FBMN, Pseudo-tripeptide, Reductase domain

## Abstract

Omicsynins are a group of pseudo-tetrapeptides produced by *Streptomyces* sp. 1647, which exhibited potent anti-influenza A virus and anti-coronavirus activities. However, its biosynthesis mechanism of C-terminus reduction remains unknown. In this work, we explored two short-chain dehydrogenase/reductase (SDR) superfamily encoding genes in the omicsynin biosynthetic gene cluster (BGC) and confirmed the necessity of *omnF*, rather than *omnG*, in the biosynthesis of omicsynins through gene deletion in vivo. Subsequently, Feature-Based Molecular Networking (FBMN) analysis revealed three pseudo-tetrapeptides with C-terminal carboxyl group and four unexpected analogues encoded by the omicsynin BGC in the *omnF* reductase (R) domain knockout mutant strain. This led to the isolation and structural characterization of a group of novel pseudo-tripeptide compounds. Compared to the known omicsynins, these pseudo-tripeptides lack the second amino acid unit and the C-terminal aldehyde group, and consequently lose their anti-coronavirus activity. In conclusion, our work highlights the effectiveness of FBMN in unveiling cryptic analogues and clearly underscores the essential role of the R domain of OmnF in the biosynthesis of the C-terminal aldehyde warhead.

**Graphical Abstract:**

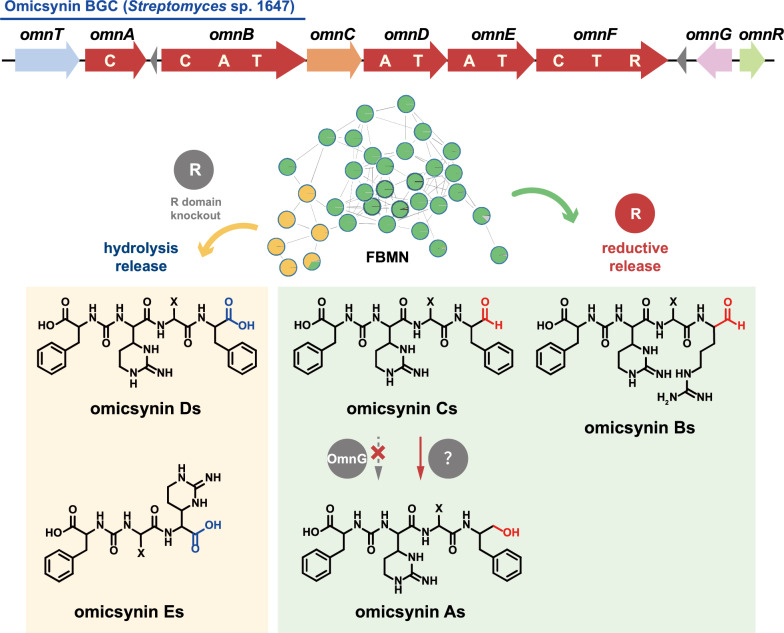

**Supplementary Information:**

The online version contains supplementary material available at 10.1007/s13659-025-00560-5.

## Introduction

Natural products, especially those derived from microorganisms, are a crucial source of clinical drugs with diverse biological activities. Within this category, a significant class with remarkable medicinal potential is synthesized through nonribosomal peptide synthetase (NRPS) mechanisms. Notable examples include well-known antibiotics like vancomycin and daptomycin [1]. Typically, NRPSs follow the co-linearity rules in their modular architecture, which is composed of several types of domains: an adenylation (A) domain that selects and activates an amino acid substrate as a building block, a thiolation (T) domain that tethers the growing peptide chain, and a condensation (C) domain that catalyzes peptide bond formation. Additionally, some optional domains, such as methyltransferases (MT), reductase (R), or epimerization (E) domains may modify the structure to form the final products [2]. However, exceptions to the co-linearity rule in NRPS can occur. For instance, module skipping happens when certain modules are bypassed during synthesis; module iteration involves using a module multiple times; and cross-module utilization occurs when modules from different NRPS pathways combine [3]. Moreover, some NRPS clusters, like those involved in sansanmycin [4] and deimino-antipain [5] biosynthesis, consist solely of discrete NRPS modules and even domains, evolutionarily allowing for more flexible and diverse product biosynthesis.

**Fig. 1:**
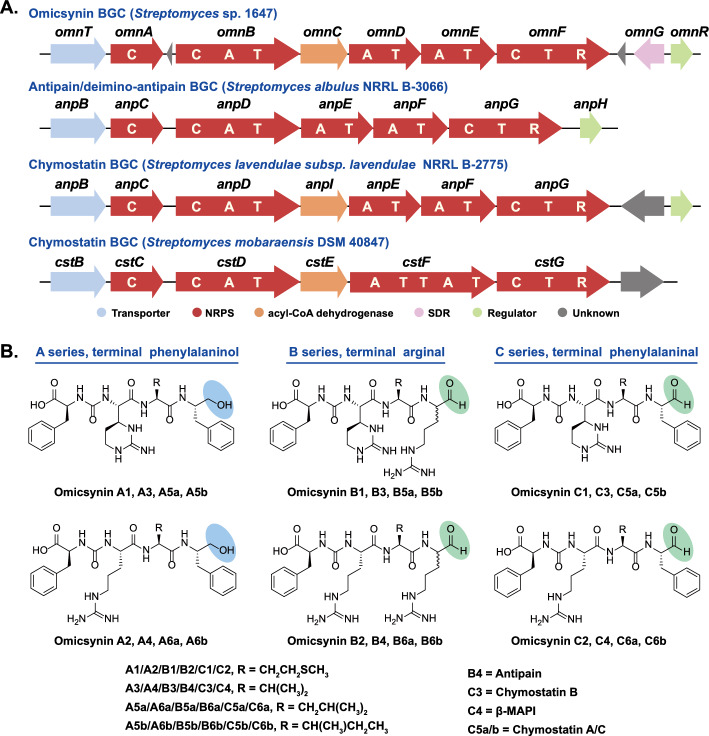
The biosynthetic gene clusters (BGCs) and chemical structures of omicsynins from *Streptomyces* sp. 1647 and the reported antipain-like compounds

In our previous study, we identified a group of pseudo-tetrapeptide secondary metabolites produced by *Streptomyces* sp. 1647, named omicsynins [6] (Fig. 1). Its biosynthetic gene cluster (BGC) was identified as a highly discrete NRPS gene cluster (Fig. 1A), including five NRPS genes with only three A domains. The BGC is generally homologous to those of deimino-antipain [5] and chymostatin [7]. However, in the omicsynin producing strain, we discovered not only antipain and chymostatin, but also more than a dozen additional compounds with diverse amino acid building blocks at the second, third and fourth positions and different C-terminal reduction states (Fig. 1B). It is remarkable that among the omicsynins identified, omicsynin B series have good anti-influenza A virus and anti-coronavirus HCoV-229E activity (EC_50_ ~ 1 μM, SI > 100), which is significantly better than antiviral drug oseltamivir phosphate and ribavirin in vitro [6]. Further research into its mechanism of action against coronaviruses has revealed that omicsynin B4 is a dual covalent inhibitor of the host proteases cathepsin L and TMPRSS2 [8]. It exerts its effect through its C-terminal aldehyde group, which binds to the cysteine and serine at the active sites of these host proteases. However, our further studies on the antiviral activity of omicsynin Bs in vivo are hindered by the difficulty in isolating and purifying sufficient quantities from the fermentation broth of the producing organism. Similar challenges arise when attempting to synthesize these compounds through chemical methods. This may be due to the C-terminal arginal of omicsynin Bs potentially forming various cyclic hemiaminals and hydrates in aqueous solution [5]. Furthermore, the largely unexplored biosynthetic pathways of this compound have hampered efforts to achieve targeted and efficient production of omicsynin Bs. Consequently, we are committed to unraveling the biosynthetic mechanisms of omicsynins, with a particular focus on the reduction process at the C-terminus.

Highly similar to the BGCs of ureido tetrapeptide aldehydes such as deimino-antipain and chymostatin, the identified omicsynin BGC (*omn*) is a discrete NRPS assembly line characterized by a conserved C-terminal reductase (R) domain (Fig. 1). The R domain has been reported to catalyze the reductive release of polyketide (PK) or nonribosomal peptide (NRP) chains as either aldehydes (via a two-electron reduction) or primary alcohols (via a four-electron reduction) [9]. Additionally, the *omn* cluster harbors three additional genes (*omnR*, *omnC*, *omnG*) compared with the reported antipain-like BGCs (Fig. 1). OmnR, a TetR family regulator, has been shown to act as a cluster-situated positive regulator, as its overexpression increases the transcriptional level of *omn* core genes [6]. OmnC is a homologue of AnpI (76% sequence identity), which was recently identified as an arginine cyclase involved in antipain biosynthesis [10]. *omnG*, a unique short-chain dehydrogenase/reductase (SDR)-encoding gene within the *omn* cluster, has not yet been investigated for its potential role in the structural diversification of omicsynins.

In this study, knockout mutants of *omnF* and *omnG* within the omicsynin BGC were constructed to investigate the mechanism responsible for formation of distinct C-terminal reduction products-either aldehyde or alcohol moiety. We also established a Feature-Based Molecular Networking (FBMN) approach to facilitate the investigation of omicsynin intermediates or shunt products within a complex metabolomic background. We identified the expected pseudo-tetrapeptides as well as unexpected pseudo-tripeptides featuring a C-terminal carboxyl group. These novel pseudo-tripeptides were further validated through isolation and structure elucidation, and their anti-coronavirus activity was determined.

## Results and discussion

### Characterization of the role of *omnG* and *omnF* in omicsynin biosynthesis

**Fig. 2:**
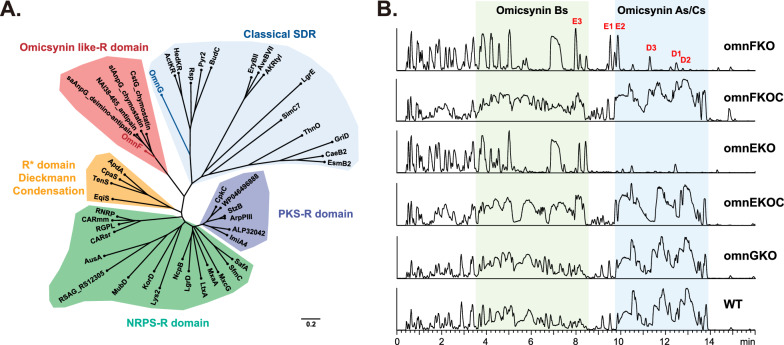
Characterization of the role of *omnG* and *omnF* in omicsynin biosynthesis. **A** Phylogenetic analysis for R domain and SDR proteins. Phylogenetic tree of the OmnG and R domain of OmnF with other SDR proteins was constructed by Neighbor-joining method with bootstrap analysis of 1000 replications in MEGA-X. **B** UPLC-( +)ESI–MS analysis of the fermentation crude extracts obtained from the wild-type (WT, *Streptomyces* sp. 1647), omnGKO, omnFKO, omnFKOC, omnEKO and omnEKOC strains. D1-D3 and E1-E3 were the new identified omicsynin analogues

Sequence analysis of OmnG and the terminal reductase (R) domain of OmnF revealed that both belong to the short-chain dehydrogenase/reductase (SDR) superfamily of NAD(P)H dependent oxidoreductases. SDR proteins exhibit diverse biochemical functions, including dehydratases, decarboxylases, reductases, isomerases and epimerases [11]. SDRs have been divided into two major families, Classical and Extended, with different Gly-motifs in the cofactor-binding regions (TGxxxGxG in classical SDRs and TGxxGxxG in extended SDRs), catalytic active sites (Sx_12_YxxxK in classical SDRs and S[S/T]…YxxxKxxxE in extended SDRs), and chain lengths (~ 250 residues in classical SDRs and ~ 350 in extended SDRs) [12, 13]. Previous reported NRPS/PKS R domains have been classified as members of the extended SDRs [9, 14, 15]. A phylogenetic analysis of homologs revealed that OmnG clusters with classical SDRs and contains the conserved NADPH-binding motif and catalytic active residues (Fig. 2A, Fig. S1). Notably, although the omicsynin-like (antipain/chymostatin) R domains harbor the conserved NADPH-binding motif and catalytic active residues characteristic of extended SDRs (Fig. S1), they possess shorter chain lengths (~ 300 residues) and show evolutionary distinction from previously characterized R domains involved in NRPS/PKS reductive release and Dieckmann condensation [16] (Fig. 2A).

Previous structural studies have shown that NRPS/PKS R domains typically are composed of an N-terminal NADPH-binding Rossmann fold with seven parallel beta sheets flanked by five alpha helices, and a less conserved C-terminal subdomain containing a helix-turn-helix (HTH) motif [9, 14, 15]. The 3D structure of the OmnF R domain complexed with NADPH was predicted using AlphaFold3. The overall structure exhibits strong architectural similarities to the NRPS/PKS-R domains (Fig. S2). Structural alignment with the NRPS-R domain of MxaA (PDB: 4U7W) [14] displays a root-mean-square deviation (RMSD) of 3.053 Å through 302 residues of the alpha carbon backbone. The most notable difference was observed in the C-terminal HTH motif. The terminal HTH motif, a distinctive region involved in substrate recognition and binding, is present in NRPS/PKS terminating R domains [9, 14, 15] but absent in the OmnF R domain. These observations suggest that the omicsynin-like R domain may exhibit distinct catalytic characteristics.

Considering bioinformatic prediction and structural features of omicsynins, we speculated that the terminal R domain of OmnF is responsible for the generation of C-terminal aldehyde group of omicsynin Bs and Cs, while OmnG likely catalyzes its reduction to a primary alcohol in omicsynin As (Fig. 1).

To validate our bioinformatic survey, the knockout mutant of *omnG* gene (omnGKO) was constructed in *Streptomyces* sp. 1647 (Fig. S3). The *omnE* knockout mutant (omnEKO) and its complementary strain (omnEKOC), which were constructed previously [6], were analyzed simultaneously. Comparative analysis of the fermentation extracts via UPLC-HR-ESI–MS revealed that a series of omicsynin-like compounds with retention times of 3.6–8.6 min and 9.8–13.9 min were completely abolished in the omnEKO but not as those in omnGKO mutant (Fig. 2B). Importantly, the proportion of A series and C series in omnGKO mutant was almost identical to those in wild-type strain (Fig. S4). This suggested that OmnG was not responsible for the alcohol group formation in omicsynin As in vivo. Then, we purified the OmnG protein (Fig. S5) and performed an in vitro enzymatic activity assay. Using chymostatin as the substrate, after incubation for 3 h at 30 °C with OmnG and NADPH, its consumption did not differ significantly from that in the negative control (the same reaction system with boiled protein), and no additional alcohol products were detected (Fig. S6). This result is consistent with the in vivo knockout experiment, further confirming that OmnG is not responsible for the formation of the terminal alcohol group in omicsynin As.

**Fig. 3:**
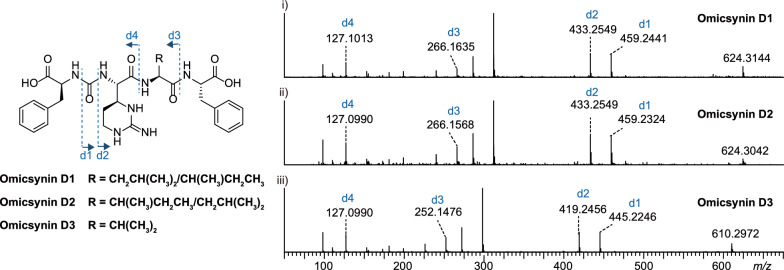
Omicsynins D1-D3 were identified based on the MS/MS fragmentation data

Considering that the results of *omnG* suggest it plays no significant role in omicsynin biosynthesis, we next aimed to explore the function of *omnF* gene. The R domain knockout mutant of *omnF* gene (omnFKO) was constructed by in-frame deletion (Fig. S7) in *Streptomyces* sp. 1647. The genetically complementary strain (omnFKOC) was obtained by reintroducing intact gene *omnF* into the mutant omnFKO to confirm that the gene knockout effect did not result from the polar effect of the deletion. Comparative analysis of the fermentation extracts revealed that a series of omicsynin-like compounds were completely abolished in the omnFKO as those in omnEKO mutant, and could be restored in the complementary strains omnFKOC (Fig. 2B). This further confirmed that OmnF was responsible for the biosynthesis of omicsynins in vivo. After a careful review of the LC–MS results, it was confirmed that the omnFKO mutant produced three putative pseudo-tetrapeptide precursors lacking carbonyl reduction (named omicsynin D1, D2, D3), which were identified based on the MS/MS fragmentation data (Fig. 3 and Fig. S8). Therefore, the R domain was demonstrated to function in the reductive release of omicsynins.

We attempted to explore whether the OmnF R domain could catalyze a two- or four-electron reduction to yield different omicsynin products in vitro. Efforts have been made to perform in vitro assays of purified OmnF protein with the surrogate substrate *S*–*N*-acetyl-cysteamine (SNAC)-tethered omicsynin B4/C4. However, only trace amounts of the corresponding aldehyde product were detected, likely because the PCP-tethered tetrapeptide is the genuine substrate (data not shown). When chymostatin was used as the substrate, the aldehyde product was not further reduced to the corresponding alcohol (data not shown). Together with the observation that OmnG is also incapable of catalyzing this reduction, these results imply that the formation of omicsynin As may be mediated by another reductase encoded outside the gene cluster. This work warrants further in-depth mechanistic investigation in the future.

### Uncovering cryptic omicsynin analogues via FBMN

Due to the substrate flexibility of A domain, non-collinearity modules, or modification by various tailoring enzymes, a single NRPS gene cluster often corresponds to multiple product structures, including some biosynthetic intermediates or shunt products. These are difficult to predict and discriminate by HPLC or even LC–MS, given the complexity of the metabolomic background. MS/MS molecular networking tools on Global Natural Product Social (GNPS) platform (https://gnps.ucsd.edu) have been used to investigate the known or unknown analogue structures based on spectral mining [17]. Feature-Based Molecular Networking (FBMN) is an advanced approach within the GNPS platform. Compared to classical molecular networking, FBMN supports quantitative analysis and enables effective resolution of isomeric compounds [18]. This approach can accelerate the discovery of unknown or unexpected metabolites associated with a target NRPS cluster, thereby facilitating a more comprehensive and in-depth understanding of its biosynthetic mechanisms.

**Fig. 4:**
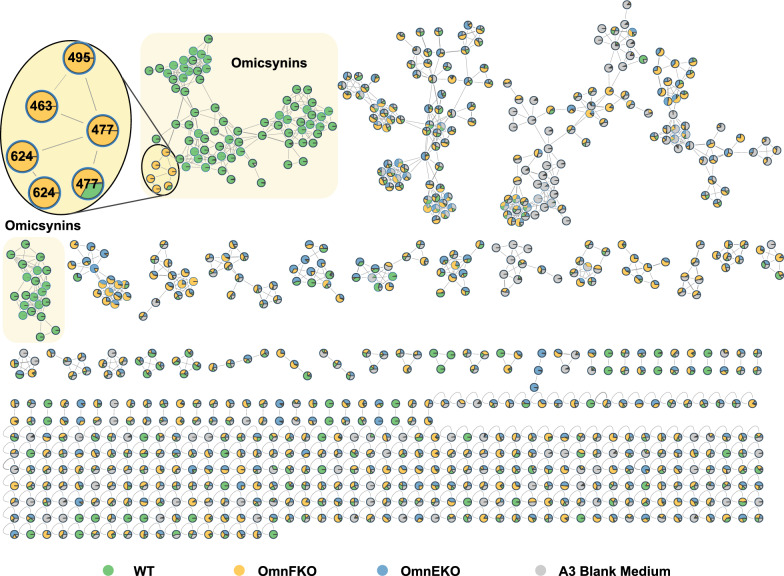
FBMN analysis based on the MS/MS fragmentation data of fermentation crude extracts of the wild-type *Streptomyces* sp. 1647, omnFKO, omnEKO and A3 medium

Intriguingly, when compared to the metabolite profile of the omnEKO strain, it appears that there are some additional metabolites present in the omnFKO strain (Fig. 2B). To efficiently identify possible *omn*-related metabolites in omnFKO strain, a FBMN analysis was applied. The UPLC-HR-ESI–MS/MS data of wild-type *Streptomyces* sp. 1647, omnEKO, and omnFKO fermentation extracts and blank control (culture medium A3) were submitted to the GNPS database for cluster analysis to construct a molecular network. As shown in Fig. 4, the four parts of metabolites were visualized in different colors and clustered into several molecular families (MFs). Obviously, omicsynins and their analogues are clustered in the MFs highlighted by yellow rectangle background, as these compounds were detected in the wild-type strain, but not in omnEKO. It’s worth noting that in omnFKO mutant, the molecular network clearly reveals four additional compounds with mass-to-charge ratios (*m/z* 477, 477, 463, 495 [M + H]^+^), which are significantly lower than those of known omicsynins. These compounds, likely to be tripeptides, were designated as omicsynins E1–E4, indicating that they may be biosynthetic shunt products derived from the *omn* pathway.

### Structural characterization of new omicsynin analogues

**Fig. 5:**
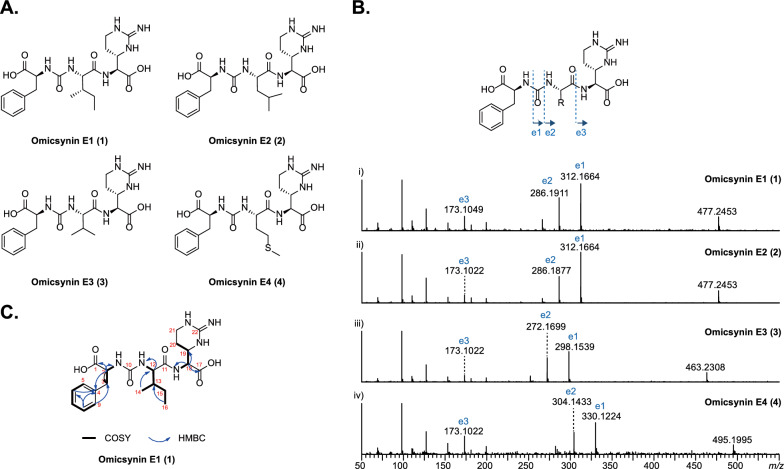
Characterization of omicsynins E1–E4. **A** The structures of **1**–**4**. **B** The MS/MS profile of **1**–**4**. **C** The 2D NMR correlations for **1**

To elucidate the structures of these unexpected omicsynin analogues, we first analyzed their MS/MS fragmentation patterns. Taking omicsynin E3 (**3**) as an example, the presence of phenylalanine (Phe) residues was confirmed by the HR-ESI–MS/MS data, which displayed a strong fragment ion at [M + H − 165]^+^, indicating the loss of a Phe residue (Fig. 5B). The characteristic fragment ions at *m/z* 272.1699 and 173.1022 indicated that the Phe residue was linked to valine (Val) residues through the ureido group. ESI–MS fragment ion at *m/z* 173.1022 were consistent with a capreomycidine (Cap) in the right C-terminal. The above data indicate that the amino acid sequence of compound **3** to be Phe–CO–Val–Cap. Comprehensive analysis of the HR-ESI–MS/MS data (Fig. 5B) of omicsynin E1 (**1**), omicsynin E2 (**2**) and omicsynin E4 (**4**) demonstrated that the Val residue in compound **3** was replaced by an isoleucine (Ile)/leucine (Leu), Leu/Ile and methionine (Met) unit, respectively. These data suggested that the amino acid sequence of pseudo-tripeptide compounds **1**–**4** are Phe–CO–Ile/Leu–Cap, Phe–CO–Leu/Ile–Cap, Phe–CO–Val–Cap, and Phe–CO–Met–Cap, respectively. This variability at the penultimate position further supported that the A domain of OmnE have relaxed substrate flexibility and capacity to generate diverse omicsynin analogues to expand the chemical diversity.

To further confirm the structures of the unexpected isomers compounds **1** and **2**, a large scale (7 L) fermentation of the omnFKO strain was then conducted. Compound **1** was obtained as a yellowish-white powder, and its molecular formula was determined as C_22_H_32_N_6_O_6_ based on positive HR-ESI–MS *m/z* 477.2450 [M + H]^+^ (calculated 477.2456, −1.3 ppm), indicating 10 degrees of unsaturation. From ^1^H − ^1^H COSY and TOCSY experiments (Fig. S11-S12), three amino acid spin systems of Phe, Ile, and Cap were determined. The assignments of the protonated carbons were obtained from the HSQC spectrum (Fig. S13), in combination with inspection of the HMBC spectrum (Table 1, Fig. 5C, Fig. S14). The NMR data analysis (Fig. S9-S14) combined with the results of secondary mass spectrometry fragmentation analysis determined that the structure of compound **1** was Phe-CO-Ile-Cap. However, due to the limited quantity of compound **2**, its structure could not be further confirmed by NMR methods. Thus, the structure of compound **2** was determined to be Phe-CO-Leu-Cap based on MS/MS fragmentation analysis and Marfey’s analysis (Fig. 5B, Fig. S15). The structures of **1** and **2** are intriguing, as they have never been identified from the homologous antipain/chymostatin biosynthetic pathway, despite being present in the wild type omicsynin producing strain.

**Table 1:** ^1^H NMR and ^13^C NMR Data for **1**

No	*δ*_H_ (*J* in Hz)	*δ*_C_, Type	No	*δ*_H_ (*J* in Hz)	*δ*_C_, Type
1	–	173.9, C	15	1.20, m; 1.43, m	24.4, CH_2_
2	4.29, td (8.0, 4.9)	54.4, CH	16	0.87, m	11.9, CH_3_
3	2.85, dd (13.8, 8.2) 3.05, dd (13.8, 5.0)	37.5, CH_2_	17	–	170.0, C
4	–	137.6, C	18	4.49, t (8.9)	54.7, CH
5	7.22, overlap	129.2, CH	19	3.24, m	52.4, CH
6	7.30, overlap	128.1, CH	20	1.68, m; 1.87, m	22.1, CH_2_
7	7.22, overlap	126.3, CH	21	3.17, m; 3.34, m	36.2, CH_2_
8	7.30, overlap	128.1, CH	22	–	154.0, C
9	7.22, overlap	129.2, CH	2-NH	6.47, d (8.0)	–
10	–	157.1, C	12-NH	8.03, d (9.0)	–
11	–	174.5, C	18-NH	6.61, d (8.7)	–
12	4.09, dd (8.9, 4.2)	59.0, CH	19-NH		–
13	1.94, m	35.9, CH	21-NH		–
14	0.87, m	16.3, CH_3_	22-NH		–

^1^H and ^13^C NMR data were recorded at 600 and 150 MHz, respectively. The assignments were based on 2D NMR (^1^H–^1^H COSY, TOCSY, HSQC, and HMBC) experiments

Then the absolute configurations of the amino acid residues in **1** and **2** were investigated using an advanced C_3_ Marfey’s analysis. The acid hydrolysates of **1**, **2** and chymostatin (used as 2*S*, 3*S*-Cap standard) were derivatized with l- or d-FDAA reagents, followed by LC–MS analysis. Comparison of the retention time of l-FDAA-derivatized and d-FDAA-derivatized 2*S*, 3*S*-Cap in chymostatin, l-Phe, l-Leu, l-Ile, and l-*allo*-Ile standards confirmed the presence of 2*S*, 3*S*-Cap, l-Ile, and l-Phe residues in **1**, 2*S*, 3*S*-Cap, l-Leu, and l-Phe residues in **2** (Fig. S15).

### Proposed biosynthesis pathway of omicsynins

**Fig. 6:**
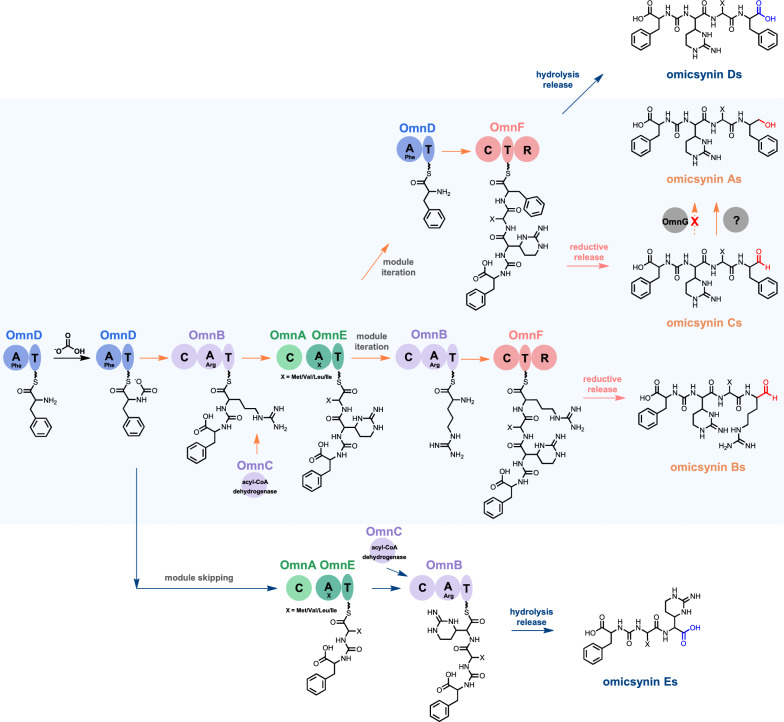
Proposed biosynthetic pathway of omicsynins

Based on genetic and chemical evidence from our current findings, together with previous reports [5, 10], we propose a preliminary biosynthetic pathway for the omicsynins (Fig. 6). Compared to the pseudo-tetrapeptide aldehyde omicsynin Bs (Fig. 1), the newly identified pseudo-tripeptide Es lack the Arg residue at the second position within the modular architecture. Thus, we propose that these unexpected pseudo-tripeptides could be installed by skipping OmnB module, which is putatively responsible for the incorporation of Arg during the biosynthetic assembly process. As expected, in the absence of OmnF R domain catalysis, omicsynin D series and E series bear a carboxyl group at the C terminus instead of an aldehyde or a primary alcohol moiety. This suggests that the R domain within OmnF is responsible for the biosynthesis of the aldehyde moiety of omicsynin Bs and Cs. However, the mechanism responsible for the formation of the alcohol moiety of omicsynin As remains unclear. Given that OmnG does not catalyze this step and the OmnF R domain also appears unlikely to participate, it is plausible that another reductase encoded outside the gene cluster mediates this transformation. Collectively, our genetic and chemical studies provide valuable insights into the biosynthetic logic underlying the structural diversity of omicsynins.

### Antiviral activity of the pseudo-tripeptides

Our previous study [6, 8] revealed that the omicsynin B series possesses potent and broad-spectrum antiviral activity, notably against influenza A virus and human coronavirus HCoV-229E. However, the pseudo-tripeptides omicsynin E1 and E2 failed to display antiviral activities at the concentration up to 200 µM against human coronavirus HCoV-229E in human hepatic Huh7 cells or Huh7.5 cells (Table S3). This result further supports the notion that the electrophilic aldehyde warhead contained in the omicsynin Bs is essential for their antiviral activity.

## Conclusions

In summary, we confirmed the necessity of *omnF*, instead of *omnG*, in the biosynthesis of omicsynins through gene deletion and complementation in vivo. We also demonstrate that FBMN provides an efficient and promising strategy to uncover cryptic analogues from complex metabolomic backgrounds, enabling effective differentiation of isomeric compounds. Based on this, three expected pseudo-tetrapeptides and four unexpected new pseudo-tripeptides were identified in OmnF R domain knockout strain. Among them, two monomers were isolated, and neither was capable of inhibiting human coronavirus HCoV-229E, supporting the crucial role of aldehyde moiety for antiviral activity. These findings suggest that the R domain within OmnF plays a crucial role in the generation of C-terminus reductions in omicsynins.

## Material and methods

### Strains, plasmids, and culture conditions

Bacterial strains, plasmids and primers used in this study were listed in Table S1 and Table S2. The fermentation and conjugation transfer conditions of *Streptomyces* sp. 1647 (China Pharmaceutical Culture Collection No. CPCC 200451) was performed as described before [6].

### Gene knockout and complementation

*omnG* gene was inactivated by substituting the core region with a thiostrepton resistance gene (Thi^r^) via homologous recombination (Fig. S3). The Thi^r^ substitute box was amplified from pIJ680 using primers thir_F/R containing *Xba*I restriction sites (Table S2). The primers for *omnG* gene disruption introduced restriction sites into the arms (*Hin*dIII and *Xba*I in the left arm, *Xba*I and *Eco*RI in the right arm), Then, flanking DNA fragments and Thi^r^ box were ligated into plasmid pKC1139 to give disruption plasmid pKC-omnGKO.

The R domain of *omnF* gene was inactivated by in-frame deletion via pOJ260 mediated double cross-over homologous recombination (Fig. S7). The primers for *omnF* disruption introduced restriction sites into the arms (*Hin*dIII and *Eco*RI in the upstream arm, *Eco*RI and *Bam*HI in the downstream arm) (Table S2). Then, flanking DNA fragments were digestion with *Hin*dIII/*Eco*RI and *Eco*RI/*Bam*HI, and were cloned into pOJ260 to yield the in-frame deletion plasmid pOJ-omnFKO.

The plasmids pKC-omnGKO and pOJ-omnFKO were introduced into *Streptomyces* sp. 1647 by conjugation from *E. coli* ET12567/pUZ8002. The single exchange mutants were screened on MS medium containing apramycin. The desired double crossover mutant omnGKO and omnFKO was selected by their apramycin-sensitive phenotype, and then confirmed by PCR (Fig. S3 and S7, Table S2).

For genetically complementary strain, the 2847 bp fragment of *omnF* was amplified from the genomic DNA of *Streptomyces* sp. 1647 by PCR with primer pairs of omnFKOC-F/R (Table S2) and was ligated into pEASY-Blunt-Zero vector. After digestion with *Nde*I and *Xba*I, the resulting fragment was cloned into pICLset, a pSET152-derived plasmid containing the constitutive promoter ermE*p to construct pL-omnF. The pL-omnF was introduced into omnFKO strain by conjugation from *E. coli* ET12567/pUZ8002. The recombinant strain omnFKOC were selected by apramycin resistant phenotype and were verified by PCR with primer pairs of *attB-Streptomyces*/pSET152 (Table S2).

### UPLC-QToF-HR-ESI–MS analysis of fermentation broth

*Streptomyces* sp. 1647 and its derivative strains were cultured in 100 mL A3 medium [6] at 28 °C for 2 days, and then 10% culture were transferred into 100 mL A3 medium for continuous fermentation at 28 °C for 5 days. The fermentation supernatants of *Streptomyces* sp. 1647 and its derivatives were concentrated six times by Waters C_18_ SPE. UPLC-TOF-HR-ESI–MS metabolite profiling was performed on a Waters Xevo G2-XS QTof spectrometer coupled to a Waters ACQUITY UPLC H-Class system. Chromatographic separation for metabolomics analysis was achieved using an ACQUITY UPLC CSH C_18_ column (1.7 μm, 2.1 mm × 50 mm) with gradient elution (solvent A, acetonitrile + 0.1% formic acid; solvent B, H_2_O + 0.1% formic acid; A/B = 10/90–30/70, v/v, over 10 min; A/B = 30/70–50/50, v/v, over 7 min; A/B = 50/50–100/0, v/v, over 5 min; flow rate = 0.3 mL/min; column temperature, 30 °C). Mass spectral data were acquired in continuum mode using the fast DDA function. ESI source parameters were set as follows: source temperature 120 °C, cone gas flow 50 L/h, desolvation temperature 450 °C, desolvation gas flow 800 L/h, capillary voltage 3 kV, sample cone voltage 40 V. Detection was performed in the positive-ion mode with an *m/z* range of 100 − 2000 Da and a survey scan time of 0.5 s, while MS/MS mode was performed over an *m/z* range of 50–2000 with a scan time of 0.1 s. The collision energy was ramped from 20 to 55 eV for low-mass analytes (100 Da) and from 40 to 75 eV for high-mass analytes (2000 Da).

### FBMN analysis on GNPS

Raw MS/MS data were analyzed using MZmine 2.51, following the feature-based molecular networking (FBMN) workflow. This process included feature detection, ADAP chromatogram building, deconvolution, isotope grouping, feature alignment, data filtering, and gap filling. The parameters used in MZmine 2.51 were consistent with those reported in previous studies [19]. The resulting spectral data file (.mgf) and the corresponding feature table (.csv), containing all detected peaks, were exported and uploaded to the GNPS platform (https://gnps.ucsd.edu) for FBMN analysis. Molecular networks were constructed using the following parameters: precursor ion mass tolerance of 0.02 Da, fragment ion mass tolerance of 0.02 Da, a minimum cosine score of 0.65, and a minimum of six matched fragment ions. The resulting networks were visualized using Cytoscape software (version 3.10.3).

### LC–MS analysis of fermentation broth

LC–MS analysis was carried out in positive full-scan mode with an Agilent 6410 Triple Quad LC–MS instrument with an *m*/*z* range of 100–1600 using the following conditions: column, SHISEIDO Capcell-Pak MG II C_18_ column (150 mm × 4.6 mm, 5 μm); solvent A, acetonitrile, solvent B, 0.1% formic acid in water; A/B = 5/95–30/70, v/v, over 30 min, A/B = 30/70–95/5, v/v, over 30 min, followed by A/B = 95/5, v/v, over 10 min; flow rate, 1.0 mL/min; column temperature, 40 °C.

### Large scale fermentation and isolation of target compounds

The fermentation broth (7 L) was centrifuged at 8000 rpm for 10 min to generate supernatant and mycelium. Isolation and purification of compounds from fermentation crudes relies on LC–MS analysis. The target compounds were enriched from the supernatant using a column of macroporous absorbent resin D4006 (2 L, 7.2 × 27 cm). After washing with 4 L of water, the column were eluted with 4 L of 20% ethanol, 50% ethanol and 100% ethanol,which were concentrated under reduced pressure to afford crude extract Fr.A to Fr.C. Fr.A (7.46 g) enriched with target compound was subjected to reversed‐phase C_18_ flash chromatography with a stepwise gradient (solvent A, methanol, solvent B, 0.1% formic acid in water; A/B = 40/60, v/v, over 30 min, A/B = 40/60–50/50, v/v, over 20 min, followed by A/B = 50/50, v/v, over 40 min; flow rate, 10 mL/min) to obtain fifteen subfractions (Fr.A1 to Fr.A15). Fr.A10-Fr.A12 with target compound was further purified by semi-preparative HPLC (Shiseido Capcell PAK Phenyl UG120 column) to yield compounds **1**–**2** (solvent A, acetonitrile; solvent B, 0.1% formic acid in water; A/B = 22/78, v/v, over 35 min; flow rate at 1.5 mL/min; compound **1**, *t*_R_ 25 min; compound **2**, *t*_R_ 27 min).

### Determination of compound configuration

50 μg of compounds **1**–**2** and chymostatin were dissolved in 100 μL of 6 N HCl and hydrolyzed at 115 °C in a sealed vial for 24 h, after which the hydrolysates were separated into two equal portions and concentrated to dryness under a stream of dry N_2_. Each portion of the hydrolysates were then treated with 1 M NaHCO_3_ (30 μL), and then derivatized respectively with l- and d-FDAA (1% solution in acetone, 40 μL) at 40 °C for 2 h, after which the reaction was neutralized with 1 M HCl (30 µL) and diluted with 400 µL 10% acetonitrile in water prior to LC–MS analysis. Authentic standards of l-Phe, l-Leu, l-Ile, and l-*allo*-Ile and 2*S*, 3*S*-Cap (from chymostatin) were derivatized with l- and d-FDAA according to the above method. The prepared derivatives were injected into the LC–MS system (Agilent 6410 Triple Quad LC–MS) that was equipped with an Agilent Zorbax SB-C3 column (5 μm, 150 × 4.6 mm, at 50 °C) eluted from 15 to 60% MeOH-H_2_O with a 5% isocratic MeCN containing 1% formic acid at a flow rate of 1 mL/min.

### Antiviral activity test

The inhibitory activity of compounds against HCoV-229E was examined by means of a cytopathic effect (CPE) inhibition assay, as described previously [6].

## Supplementary Information

Supplementary material 1.

## Data Availability

The original contributions presented in the study are included in the article/supplementary material, further inquiries can be directed to the corresponding author.
