# Supplementary material for: Unraveling the metabolic potential and roles of reductases in the omicsynin biosynthetic gene cluster
Source: Nat Prod Bioprospect. 2026 Jan 9;16(1):7. doi: 10.1007/s13659-025-00560-5 (PMC12783407; doi:10.1007/s13659-025-00560-5)
Supplement: Supplementary file 1 — Supplementary material 1. [file 13659_2025_560_MOESM1_ESM.docx]

**Unraveling the Metabolic Potential and Roles of Reductases in the Omicsynin Biosynthetic Gene Cluster**

Yihong Li ^1,2,#^, Jie Fu ^1,2,#^, Hongmin Sun ^1,2^, Yu Du ^2^, Shuyi Si ^2^, Yuhuan Li ^2,3^, Xingxing Li ^1,2,^*, Jiandong Jiang ^2,3,^*, Bin Hong ^1,2,4,^*

^1^ CAMS Key Laboratory of Synthetic Biology for Drug Innovation, Institute of Medicinal Biotechnology, Chinese Academy of Medical Sciences & Peking Union Medical College, No.1 Tiantan Xili, Beijing 100050, China.

^2^ NHC Key Laboratory of Biotechnology for Microbial Drugs, Institute of Medicinal Biotechnology, Chinese Academy of Medical Sciences & Peking Union Medical College, No.1 Tiantan Xili, Beijing 100050, China.

^3^ CAMS Key Laboratory of Antiviral Drug Research, Institute of Medicinal Biotechnology, Chinese Academy of Medical Sciences & Peking Union Medical College, No.1 Tiantan Xili, Beijing 100050, China.

^4^ State Key Laboratory of Bioactive Substances and Functions of Natural Medicines, Institute of Medicinal Biotechnology, Chinese Academy of Medical Sciences & Peking Union Medical College, No.1 Tiantan Xili, Beijing 100050, China.

^#^ Yihong Li and Jie Fu contributed equally to this work.

^*^ Corresponding author.

*E-mail address*: binhong69@hotmail.com, hongbin@imb.pumc.edu.cn (B. Hong); jiang.jdong@163.com (J. Jiang); xingxingsf@163.com, lixingxing@imb.pumc.edu.cn (X. Li).

**Contents**

[Supplementary Methods 3](#_Toc211113213)

[1. Expression and purification of OmnG 3](#_Toc211113214)

[2. In vitro assay of OmnG 3](#_Toc211113215)

[Supplementary Tables 5](#_Toc211113216)

[Table S1. Strains and plasmids used in this study. 5](#_Toc211113217)

[Table S2. The PCR primers used in this study. 7](#_Toc211113218)

[Table S3. Anti-coronavirus HCoV-229E activity of omicsynin E1 and E2. 8](#_Toc211113219)

[Supplementary Figures 9](#_Toc211113220)

[Figure S1. Multiple Sequence Alignment Analysis. 9](#_Toc211113221)

[Figure S2. Structure of the R domain of OmnF and MxaA. 10](#_Toc211113222)

[Figure S3. Disruption of *omnG* gene in omicsynin BGC. 11](#_Toc211113223)

[Figure S4. HPLC-MS analyses of the compound production by omnGKO. 12](#_Toc211113224)

[Figure S5. Expression and purification of OmnG. 13](#_Toc211113225)

[Figure S6. In vitro enzyme assay of OmnG 14](#_Toc211113226)

[Figure S7. Disruption of *omnF* gene in omicsynin BGC. 15](#_Toc211113227)

[Figure S8. The HR-ESI-MS data of omicsynin D1-D3 and E1-E4. 16](#_Toc211113228)

[Figure S9. ^1^H NMR spectrum of omicsynin E1 (**1**) in DMSO-*d*_6_ (600 MHz). 17](#_Toc211113229)

[Figure S10. ^13^C NMR spectrum of omicsynin E1 (**1**) in DMSO-*d*_6_ (150 MHz). 18](#_Toc211113230)

[Figure S11. ^1^H-^1^H COSY spectrum of omicsynin E1 (**1**). 19](#_Toc211113231)

[Figure S12. TOCSY spectrum of omicsynin E1 (**1**) 20](#_Toc211113232)

[Figure S13. HSQC spectrum of omicsynin E1 (**1**). 21](#_Toc211113233)

[Figure S14. HMBC spectrum of omicsynin E1 (**1**). 22](#_Toc211113234)

[Figure S15. Marfey’s analysis of omicsynin E1 (**1**) and E2 (**2**) 23](#_Toc211113235)

[Reference 24](#_Toc211113236)

# Supplementary Methods

## Expression and purification of OmnG

The coding region of *omnG* was cloned into the *Bam*HI - *Xho*I sites of pET-28a-SUMO to give pET28a-SUMO-omnG, and then was transformed into *E. coli* BL21(DE3) competent cells (TransGen Bioscience Inc. China) for protein expression. BL21(DE3) strain was cultured in LB medium with 50 μg/mL kanamycin at 37 °C until an OD_600_ of 0.6. Then isopropyl-*β*-D-thiogalactopyranoside (IPTG) was added to the final concentration of 0.1 mM, and the cultures were incubated at 28 °C for overnight to induce expression of the recombinant His-SUMO-tagged protein. The bacteria were harvested by centrifugation (10,000× g, 10 min, 4 °C), washed twice and resuspended with binding buffer (20 mM Na_3_PO_4_, 0.5 M NaCl, 5 mM imidazole, pH 7.4). After ultrasonication, cellular debris was removed by centrifugation (12,000× g, 15 min, 4 °C). His-SUMO-tagged OmnG was then purified using HisTrap™ HP column (GE Healthcare) according to the manufacturer's instructions, eluted with elution buffer (20 mM Na_3_PO_4_, 0.5 M NaCl, 500 mM imidazole, pH 7.4) and dialyzed against SUMO-tag cleavage buffer (50 mM Tris-HCl, 1 mM DTT, 150 mM NaCl, pH 7.4) by the PD10 Desalting Columns (GE Healthcare).

To generate tag-free OmnG, 3.5 mL purified His-SUMO-OmnG was mixed with 7 μL Ulp1 (5 U/μL, Solarbio Bioscience Inc. China) and incubated for 22  h at 4°C. The mixture was loaded onto HisTrap™ HP column (GE Healthcare) to separate tag-free OmnG from His-SUMO and His-SUMO-OmnG. The flowthrough was collected to obtain tag-free OmnG and stored at -80 °C. The concentration of purified OmnG was determined using Bradford Assay Kit (Thermo Scientific). The purity of OmnG was assessed by SDS-PAGE analysis.

## *In vitro* assay of OmnG

In a volume of 50 μL reaction system, the following were contained: 50 mM Tris-HCl (pH 7.4), 2 μM OmnG, 10 mM NADPH (Sigma), 2 mM TCEP (Sigma), 10 mM MgCl_2_, and 60 μg/mL chymostatin (Sigma, cat#C7268). The same reaction system with boiled protein (100 °C in metal bath for 10 min) was used as negative control. The *in vitro* assays were carried out in 30 °C water bath for 3 h. To terminate the reaction, a two-fold volume of cold methanol was added into each reaction mixture and cooled in an ice bath for 5 min. After removal of the denatured protein by centrifugation, the reaction mixtures were filtered with microporous membrane of 0.22 μm, and then were subjected to LC-MS analysis.

LC-MS analysis of the *in vitro* enzyme reaction samples was performed in positive full-scan mode using an Agilent 6410 Triple Quadrupole LC-ESI-MS instrument with an *m*/*z* range of 50–1000. Separation was achieved on a CAPCELL PAK ADME column (4.6 × 250 mm, 5 μm). The mobile phase consisted of solvent A, 0.1% formic acid in water; solvent B, acetonitrile, with a flow rate of 1.0 mL/min. The gradient elution program was as follows: A/B = 99/1 → 80/20, v/v, over 10 min; A/B = 80/20 → 40/60, v/v, over 15 min; A/B = 40/60 → 5/95, v/v, over 5 min; A/B = 5/95 → 99/1, v/v, over 2 min; followed by A/B = 99/1, v/v, held for 13 min.

# Supplementary Tables

## Table S1. Strains and plasmids used in this study.

| **Strain/plasmid** | **Relevant characteristic** | **References** |
| --- | --- | --- |
| **Strain** |  |  |
| *Streptomyces* sp. 1647 | A strain of *Streptomyces* with high anti-influenza A virus activity | [1] |
| omnEKO | *omnE* gene deletion mutant in *Streptomyces* sp. 1647 | [1] |
| omnEKOC | *omnE* genetically complementary strain of omnEKO, Am^r^ | [1] |
| omnFKO | *omnF* gene deletion mutant in *Streptomyces* sp. 1647 | This study |
| omnFKOC | *omnF* genetically complementary strain of omnFKO, Am^r^ | This study |
| omnGKO | *omnG* gene deletion mutant in *Streptomyces* sp. 1647, Thi^r^ | This study |
| *E. coli* T1 | General cloning host | TransGen |
| *E. coli* DH5α | General cloning host | TransGen |
| ET12567/pUZ8002 | Strain used for *E. coli*/*Streptomyces* sp. 1647 conjugation, Cm^r^, Km^r^ | [2] |
| *E. coli* BL21(DE3) | Strain used for protein expression | TransGen |
| BL21(DE3)/pET28a-SUMO-omnG | Strain used for the expression of His-SUMO-omnG | This study |
| **Plasmid** |  |  |
| pEASY-Blunt-Zero | PCR fragment cloning vector, Amp^r^/Km^r^ | TransGen |
| pSET152 | A *Streptomyces* integrative vector that can be used for conjugative transfer, containing ΦC31 integration site, Am^r^ | [3] |
| pICLset | pSET152 derivative plasmid containing the constitutive promoter *ermE**p, Am^r^ | [4] |
| pL-omnF | pICLset derivative plasmid containing 2847 bp coding region of gene *omnF*, Am^r^ | This study |
| pOJ260 | *E. coli* vector, nonreplicating in *Streptomyces*, Am^r^ |  |
| pOJ-omnFKO | The pOJ260 derivative for disruption of *omnF* gene, Am^r^ | This study |
| pKC1139 | *E. coli*/*Streptomyces* shuttle vector with temperature-sensitive replication origin, Am^r^ | [3] |
| pKC-omnGKO | The pKC1139 derivative for disruption of *omnG* gene, Am^r^ | This study |
| pET-28a(+) | *E. coli* expression vector, Km^r^ | Novagen |
| pET28a-SUMO-omnG | pET28a derivative plasmid containing His-SUMO fused OmnG, Km^r^ | This study |

Am^r^: apramycin resistance; Cm^r^: chloramphenicol resistance; Km^r^: kanamycin resistance; Thi^r^: thiostrepton resistance.

## Table S2. The PCR primers used in this study.

| **Name** | **Sequence (5’-3’)*** | **Purpose** |
| --- | --- | --- |
| omnGKO_AF | ATaagcttACCCAGATCCAGAACAGCG | Used for amplifying upstream arm used in knockout of *omnG* gene. |
| omnGKO_AR | ATtctagaGACCTTGCCCTCGATTCCT |  |
| omnGKO_BF | ATtctagaAAGACGGCGGTGAAGGTG | Used for amplifying downstream arm used in knockout of *omnG* gene. |
| omnGKO_BR | ATgaattcCTGCGAGCGGATCCTCTT |  |
| Thir_F | GAtctagaTGATCATCACTGACGAATCG | Used for amplifying thiostrepton resistance gene. |
| Thir_R | GAtctagaAGGCGAATACTTCATATGCG |  |
| omnGKO-Check-P1 | GTCTTCGTCAGCAGCATCG | Used for verifying the gene *omnG* knockout strain. |
| omnGKO-Check-P2 | TGGGGAAGTTGCGGTAGAG |  |
| omnGKO-Check-P3 | CTACGAGTAGTACCTGTGCAG |  |
| omnGKO-Check-P4 | GATGTCCTGGTCGCTGTAGA |  |
| omnGKO-Check-P5 | GGGGACTTCACGACGTAGG |  |
| omnGKO-Check-P6 | CTCCATCTCGCCGGGAAG |  |
| omnFKO_AF | CGaagcttGAGCTCACCCCGATCCAG | Used for amplifying upstream arm used in knockout of *omnF* gene. |
| omnFKO_AR | TAgaattcGACGGTGCCGGTGCGGGGGC |  |
| omnFKO_BF | TAgaattcGACCAGCTCCGCTGGGGCTC | Used for amplifying downstream arm used in knockout of *omnF* gene. |
| omnFKO_BR | TAggatccGTCCAGGGTGAGGTCCAG |  |
| omnFKO-Check-P1 | TTCCCCTGGCTCGAACTG | Used for verifying the gene *omnF* knockout strain. |
| omnFKO-Check-P2 | AGGCTCAAGATTCGCTCAGA |  |
| omnFKO-Check-P3 | CCGTCCAACTCGCCGAAC |  |
| omnFKO-Check-P4 | GACGAGGAGGGTGGTACG |  |
| omnFKO-Check-P5 | GATCCACTCCGCCTGCAC |  |
| omnFKO-Check-P6 | GAGGATCCGCTCGCAGTC |  |
| omnFKOC-F | TAcatatgATGAGCGCCGTGGACCAGGC | Used for amplifying the gene *omnF.* |
| omnFKOC-R | GAtctagaTCAGCCGACCATGGCGGCCA |  |
| *attB-Streptomyces* | CGGTGGGGGTGCCAGGG | Used for amplifying 1.6 kb pSET152-*attB* integration site. |
| pSET152 | TTCGGCGGCTTCAAGTTCGG |  |
| OmnG-SUMO-F | TAggatccGTGCCAGGAATCGAGGGCAA | Used for amplifying His-SUMO-OmnG |
| OmnG-SUMO-R | TActcgagTCAGGCCTGGGCGGTGGGGC |  |

* The oligonucleotides introducing restriction sites are underlined.

## Table S3. Anti-coronavirus HCoV-229E activity of omicsynin E1 and E2.

| Compound | HCoV-229E / Huh7 | | | HCoV-229E / Huh7.5 | | |
| --- | --- | --- | --- | --- | --- | --- |
|  | CC_50_ ^a^  (*µ*M) | EC_50_ ^a^  (*µ*M) | SI ^b^ | CC_50_ ^a^  (*µ*M) | EC_50_ ^a^  (*µ*M) | SI ^b^ |
| omicsynin E1 | > 200.0 | > 200.0 | - | > 200.0 | > 200.0 | - |
| omicsynin E2 | > 200.0 | > 200.0 | - | > 200.0 | > 200.0 | - |
| omicsynin B4 | > 200.0 | 7.41 | > 27.0 | > 200.0 | 3.29 | > 60.8 |
| Nirmatelvir | > 200.0 | 0.37 | > 540.5 | > 200.0 | 1.07 | > 186.9 |

^a^ CC_50_, 50% cytotoxic concentration. EC_50_, 50% effective concentration. CC_50_ and EC_50_ were determined by CPE inhibition assay.

^b^ The SI value was calculated as the ratio of CC_50_ /EC_50_.

# Supplementary Figures


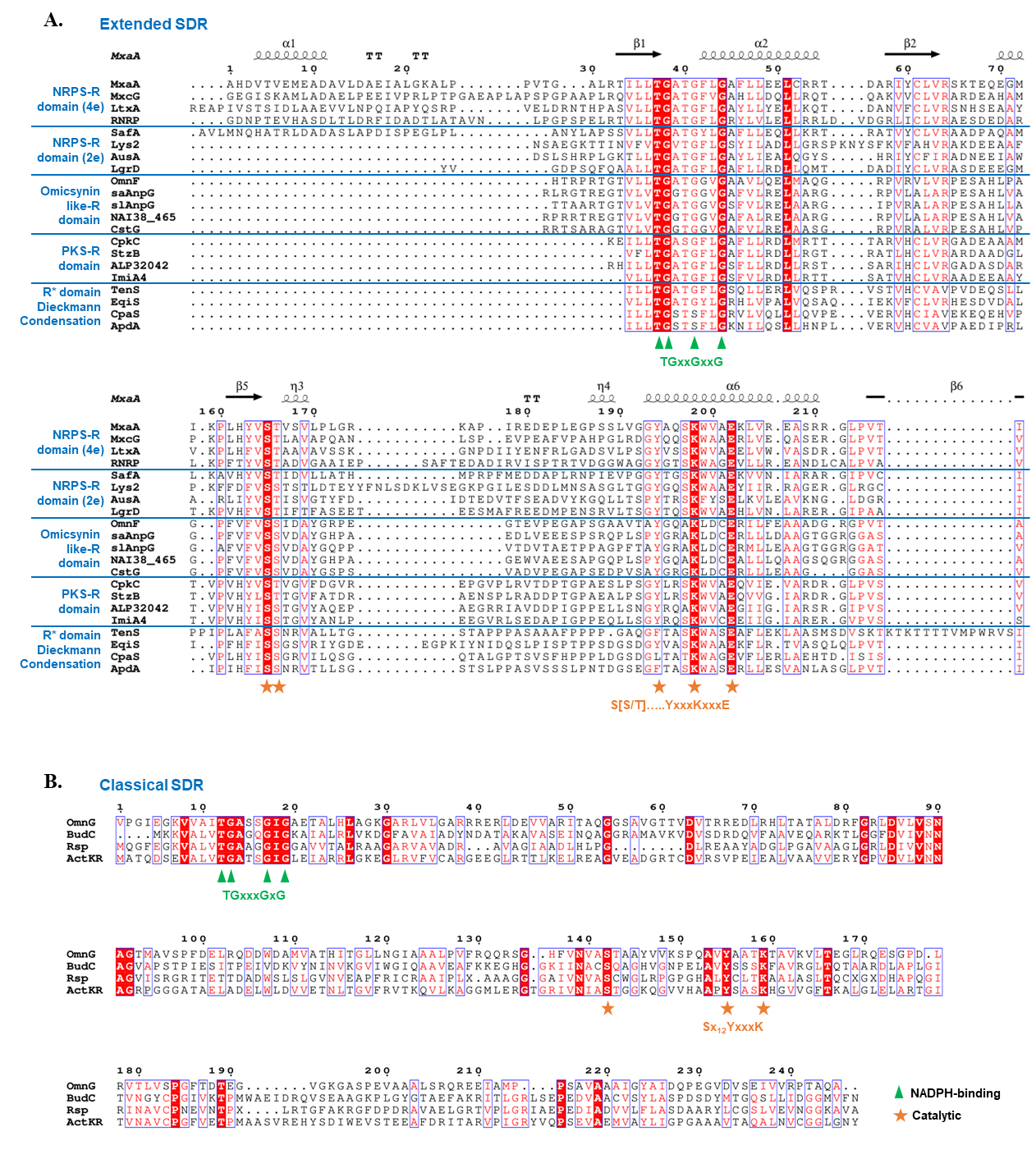


## Figure S1. Multiple Sequence Alignment Analysis.

(A). Amino acid sequence multi-alignment of the OmnF R domain with extended SDR family proteins. The sequences were annotated with corresponding secondary structures in MxaA (PDB ID: 4U7W). Arrows represented β-strands and helices represented α-helices. (B). Amino acid sequence multi-alignment of OmnG with classical SDR family proteins. The conserved residues were colored in red background. The green triangle highlights the conserved NADPH binding sites. The orange star indicates the canonical catalytic residues.


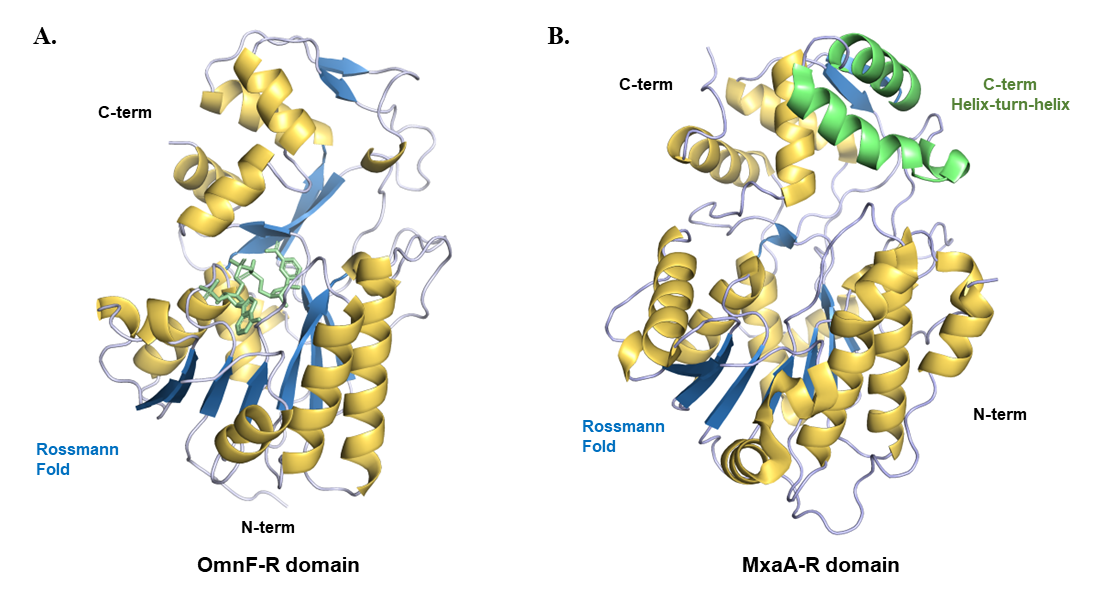


## Figure S2. Structure of the R domain of OmnF and MxaA.

(A). The 3D structure of the OmnF R domain complexed with NADPH was predicted via AlphaFold3. NADPH was colored in light green. (B). The 3D structure of MxaA R domain (chain B of PDB ID: 4U7W). The C-terminal helix-turn-helix motif was highlighted in green.


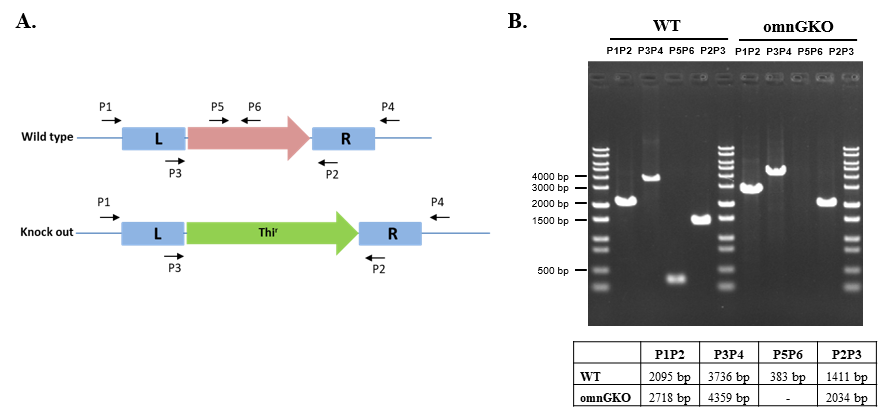


## Figure S3. Disruption of *omnG* gene in omicsynin BGC.

(A). Schematic representation for the PCR verification. (B). PCR analysis of the *omnG* knockout mutant omnGKO and the wild type strain (*Streptomyces* sp. 1647) were carried out with primers listed in Table S2.


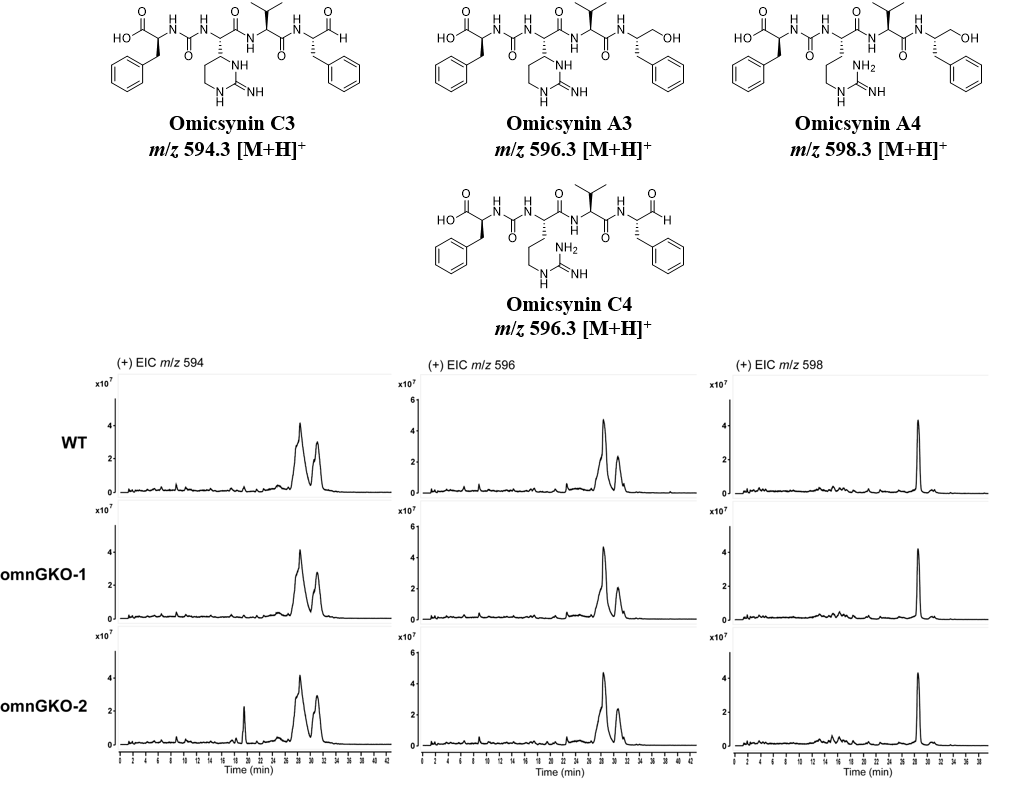


## Figure S4. HPLC-MS analyses of the compound production by omnGKO.

Extracted ion chromatogram (EIC) of omicsynin C3/A3/C4/A4 from the *omnG* knockout mutant omnGKO (two clones) and the wild type strain (WT, *Streptomyces* sp. 1647).


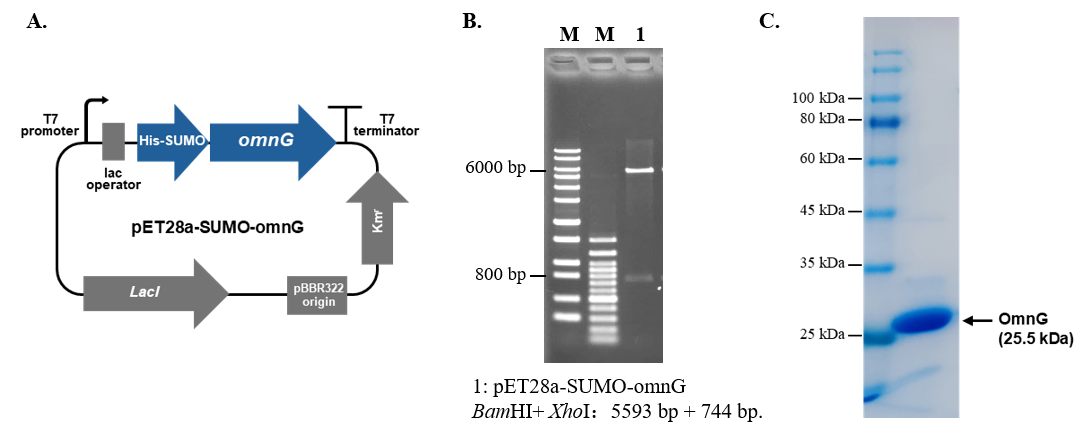


## Figure S5. Expression and purification of OmnG.

(A). Schematic representation for *omnG* expression plasmid. (B). Restriction analysis of expression plasmid. Digestion of pET28a-SUMO-omnG with *Bam*HI and *Xho*I. M, DNA marker. (C). SDS-PAGE analysis of OmnG protein. The OmnG protein was purified and visualized by Coomassie Brilliant Blue staining after SDS-PAGE on 10 % polyacrylamide gel. The theoretical molecular weight of OmnG is 25.5 kDa.


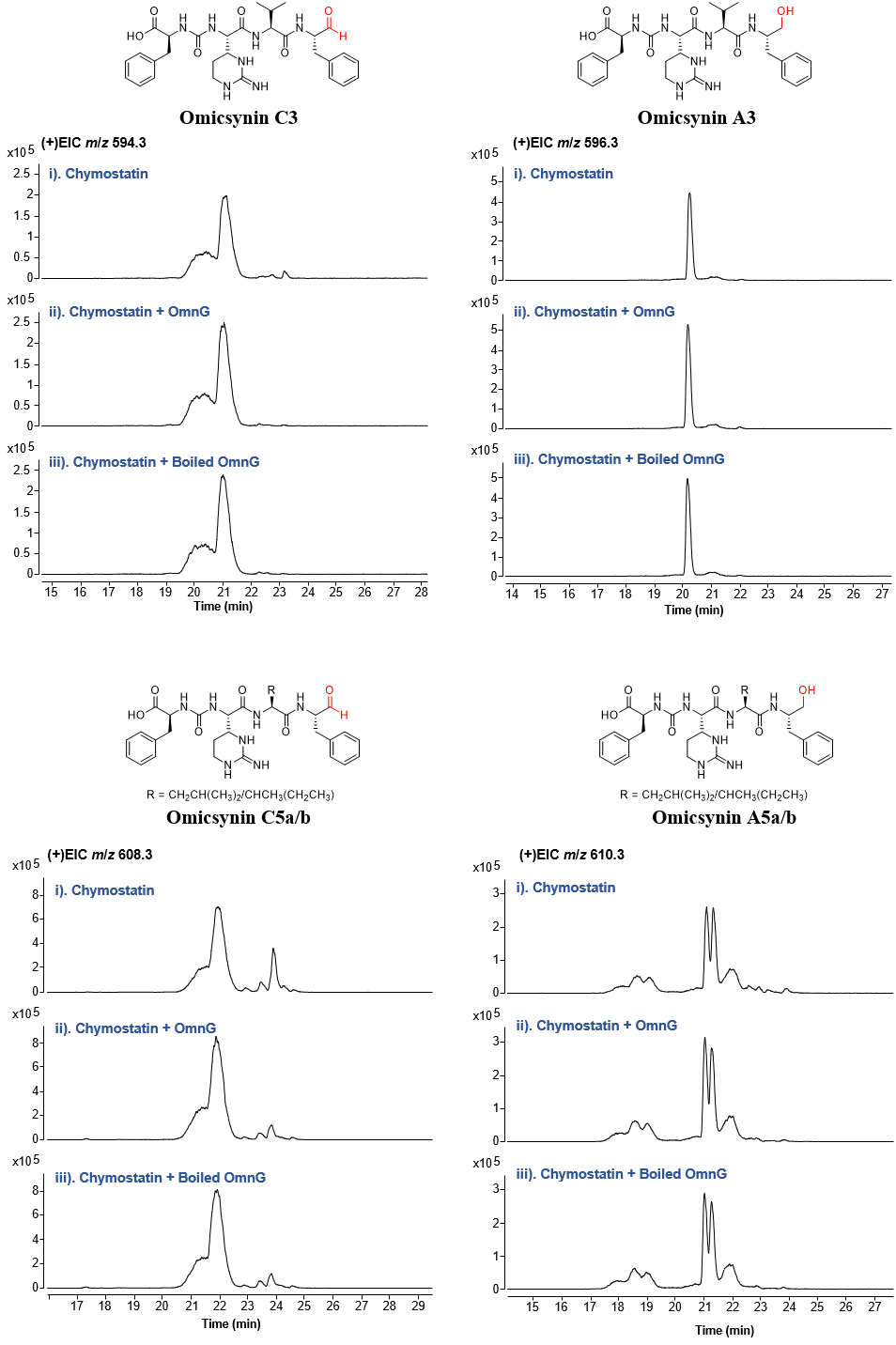


## Figure S6. *In vitro* enzyme assay of OmnG

LC-MS analysis of *in vitro* assay of OmnG with chymostatin (ii). The same reaction system with boiled OmnG was used as negative control (iii). Chymostatin standard (i) from Sigma (cat#C7268) is a mixture comprising three major components (chymostatin B/A/C) equivalent to our omicsynin C3/C5a/C5b. Although not specified by the supplier, our mass spectrometric analysis revealed minor components corresponding to the terminal phenylalaninol forms of omicsynin A3/A5a/A5b.


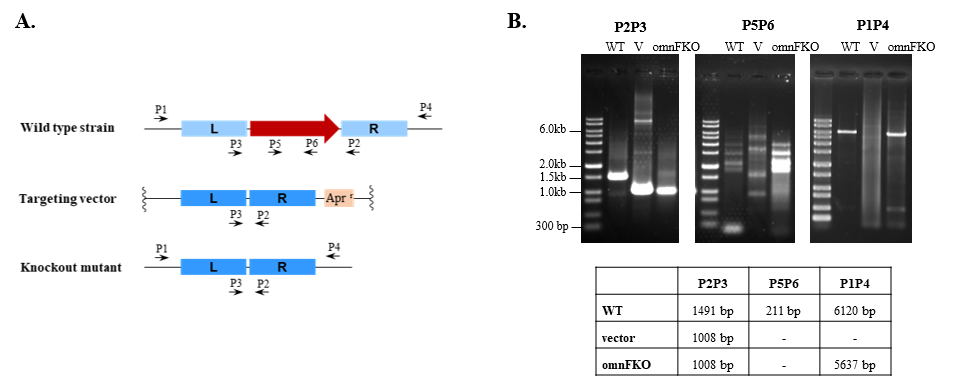


## Figure S7. Disruption of *omnF* gene in omicsynin BGC.

(A). Schematic representation for the PCR verification. (B). PCR analysis of the *omnF* knockout mutant omnFKO, the wild type strain (WT, *Streptomyces* sp. 1647) and deletion plasmid pOJ-omnFKO (V) were carried out with primers listed in Table S2.


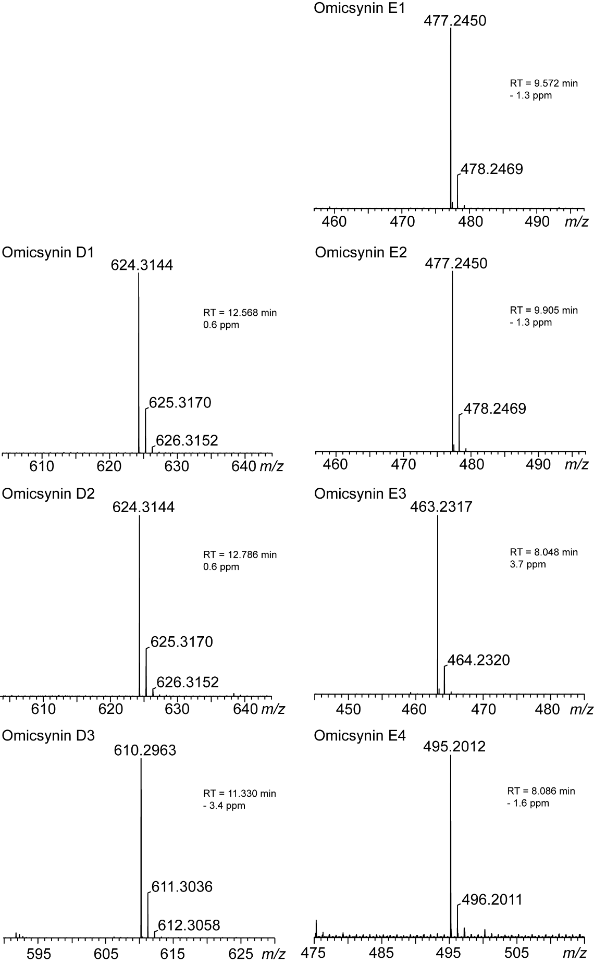


## Figure S8. The HR-ESI-MS data of omicsynin D1-D3 and E1-E4.


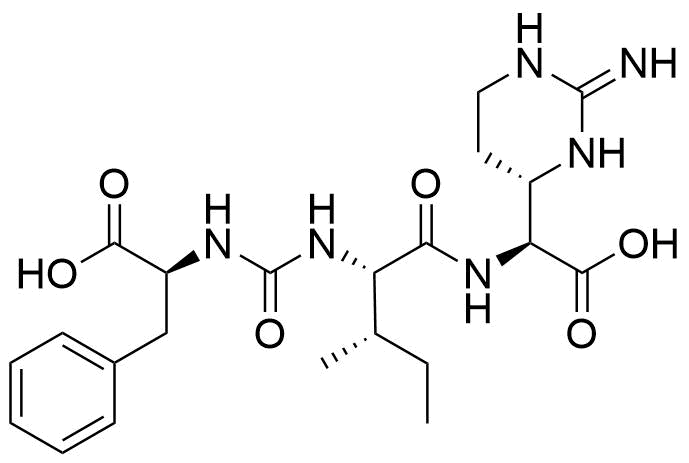

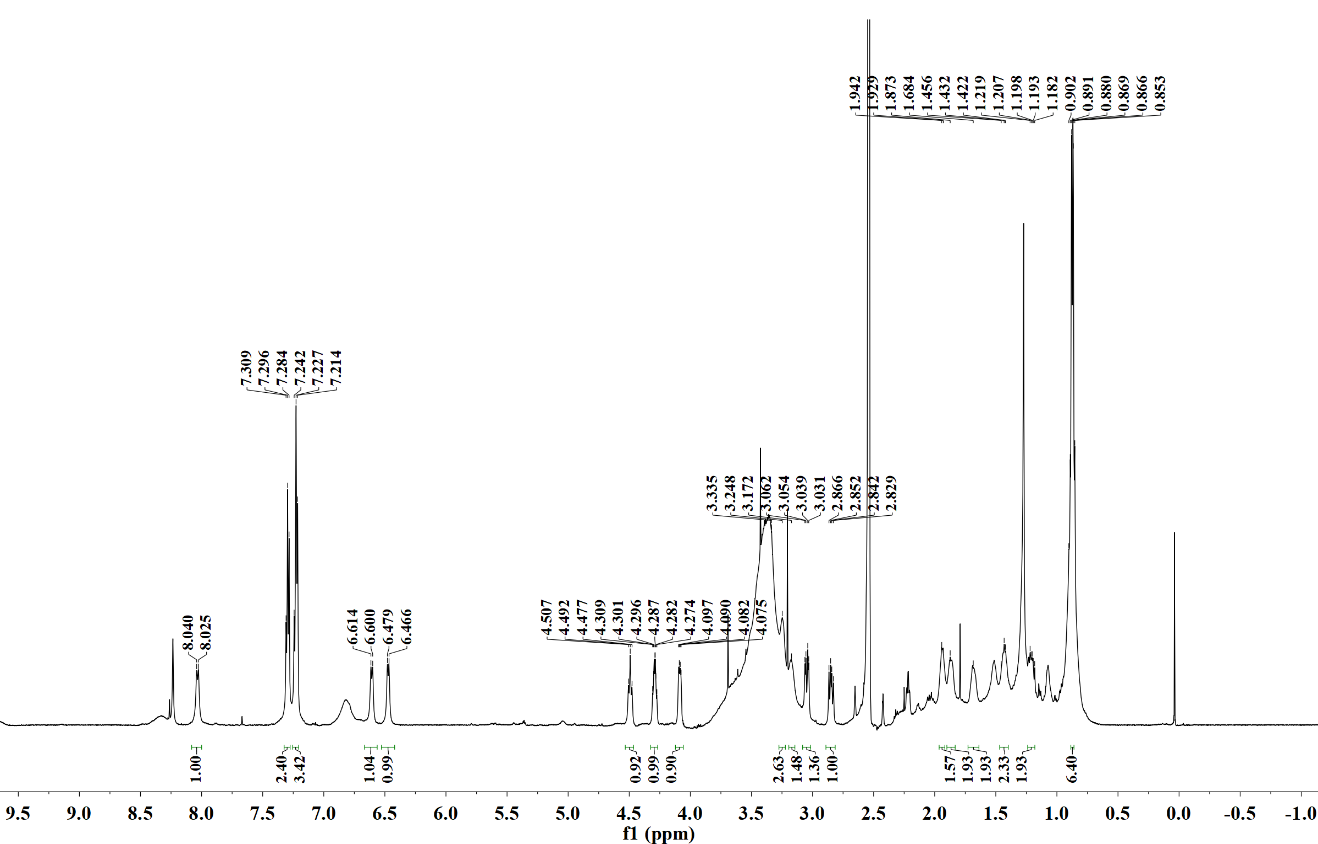


**Figure S9. ^1^H NMR spectrum of omicsynin E1 (1) in DMSO-*d*_6_ (600 MHz).**


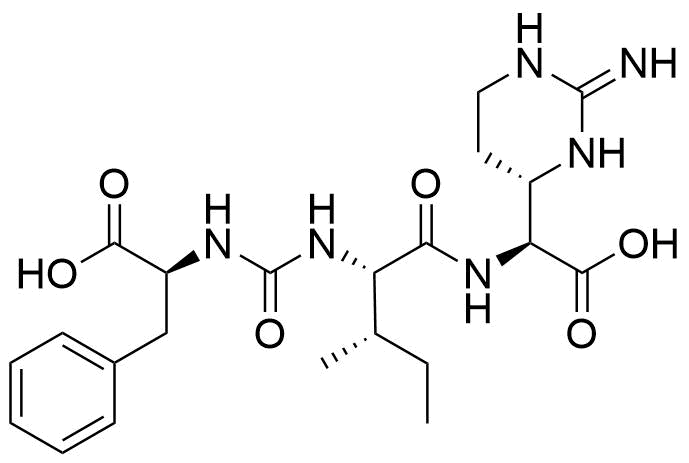

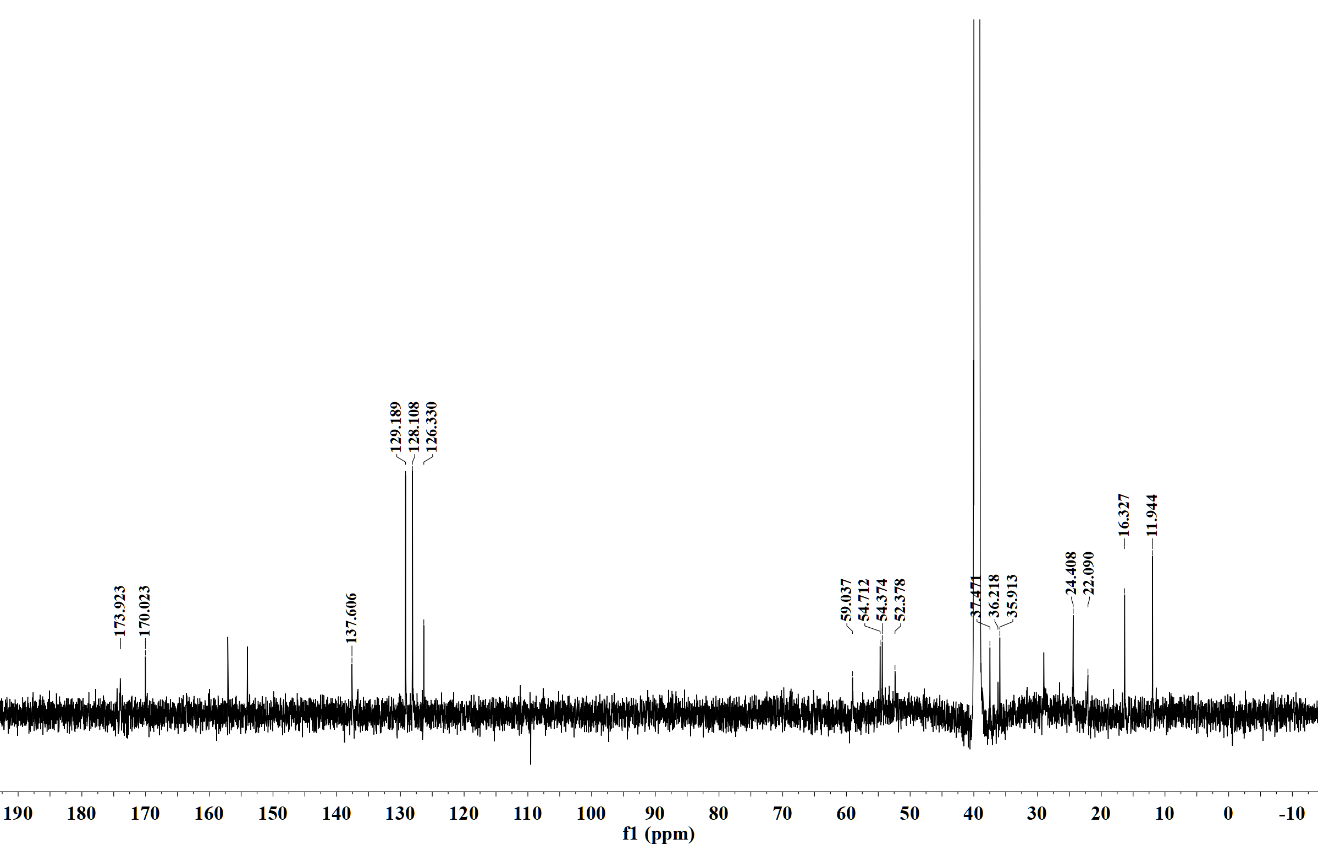


**Figure S10. ^13^C NMR spectrum of omicsynin E1 (1) in DMSO-*d*_6_ (150 MHz).**


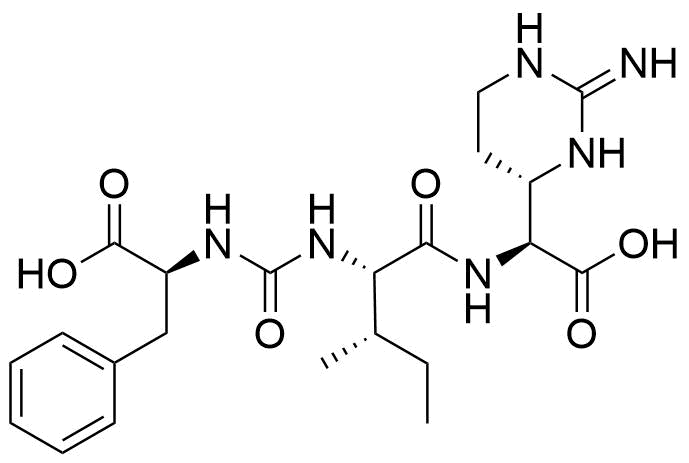

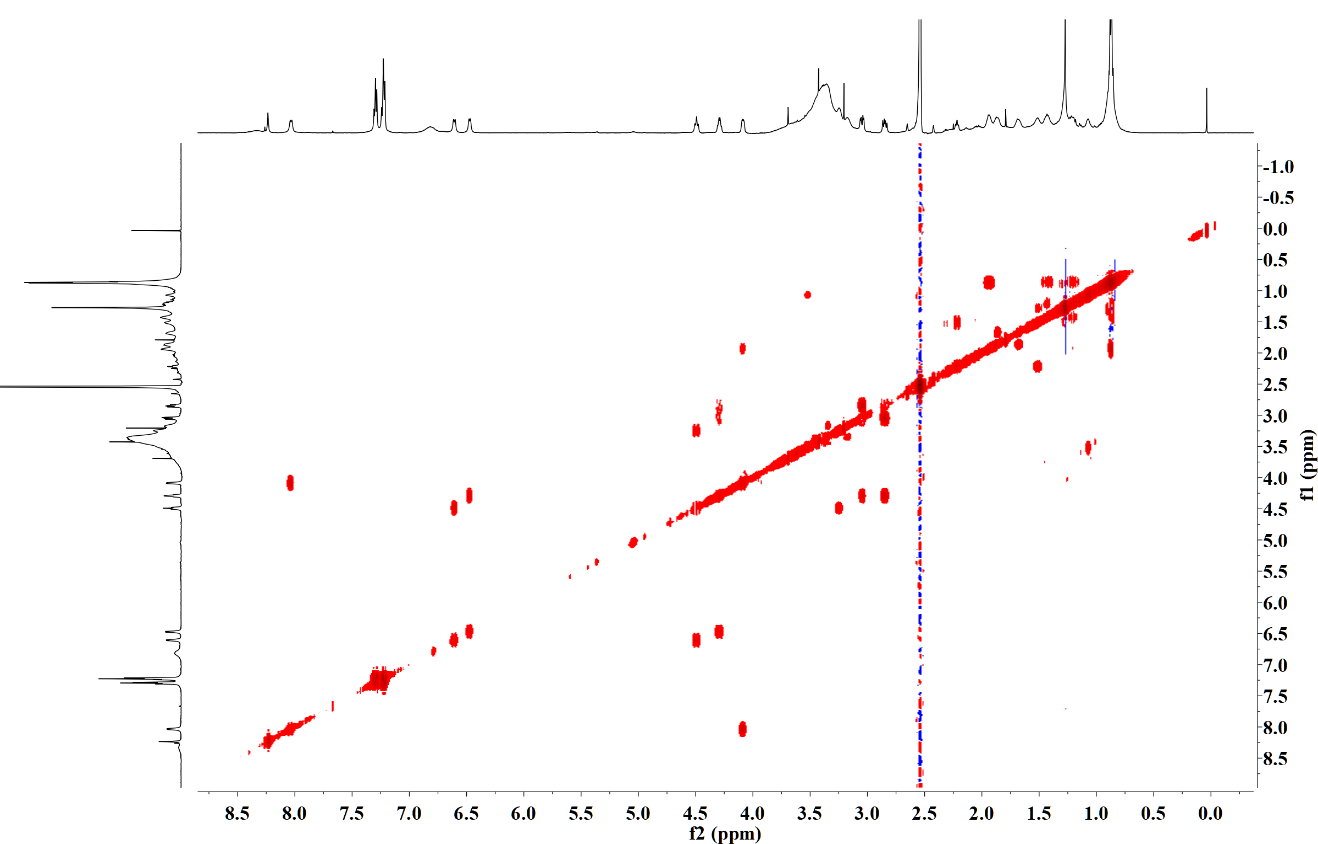


**Figure S11. ^1^H-^1^H COSY spectrum of omicsynin E1 (1).**


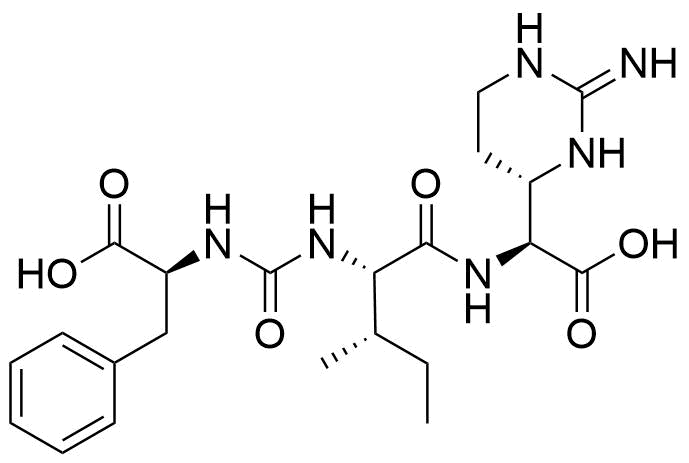

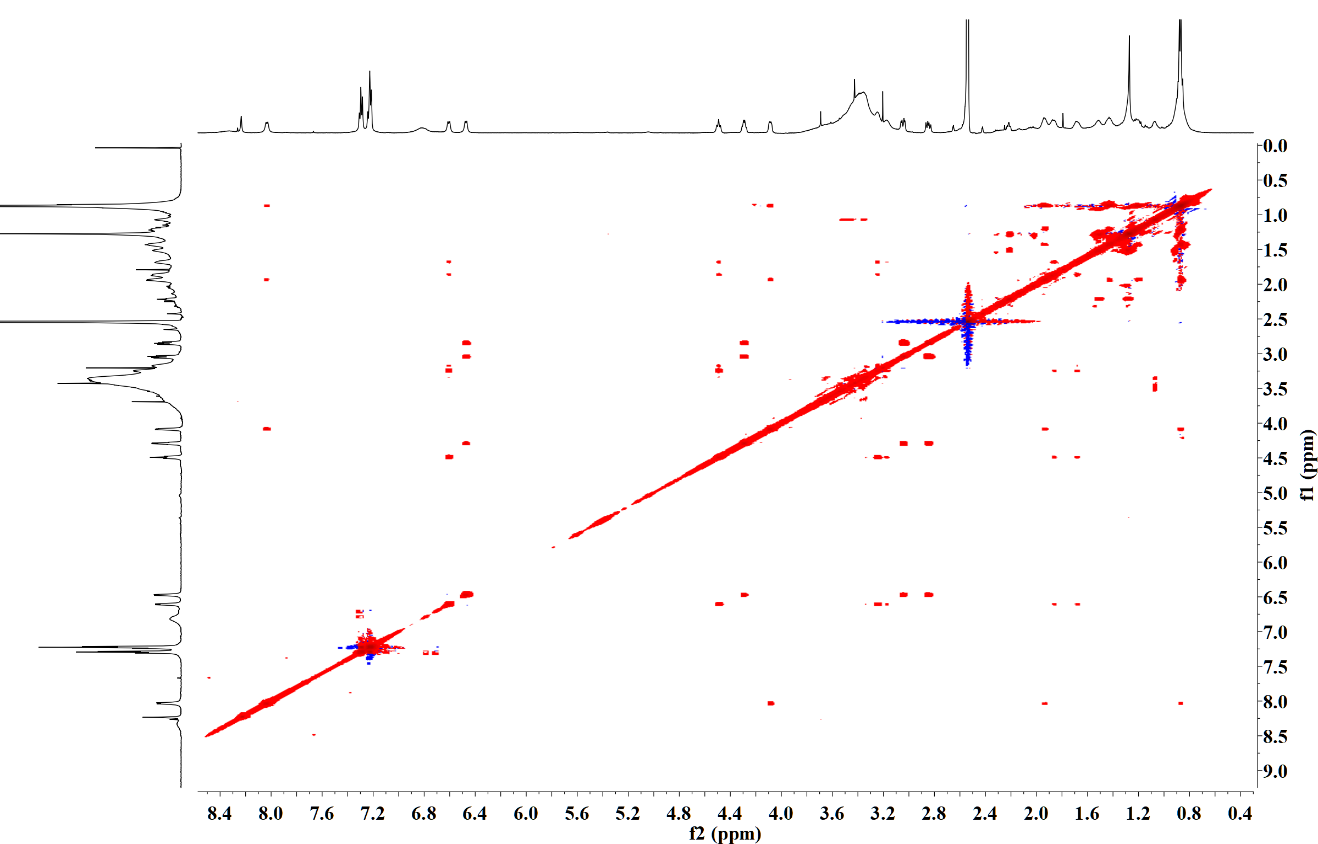


**Figure S12. TOCSY spectrum of omicsynin E1 (1)**


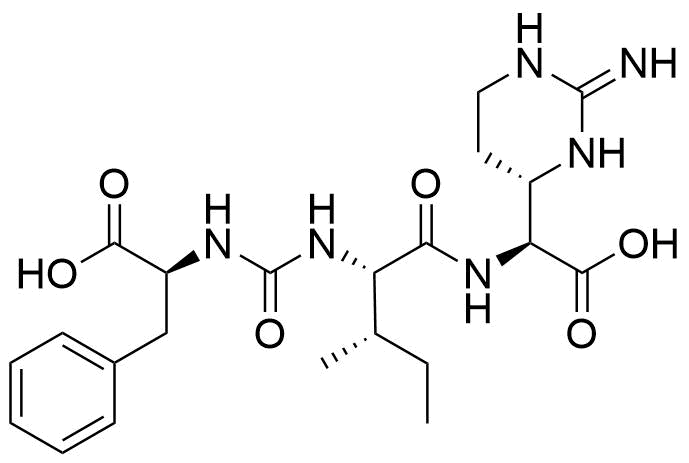

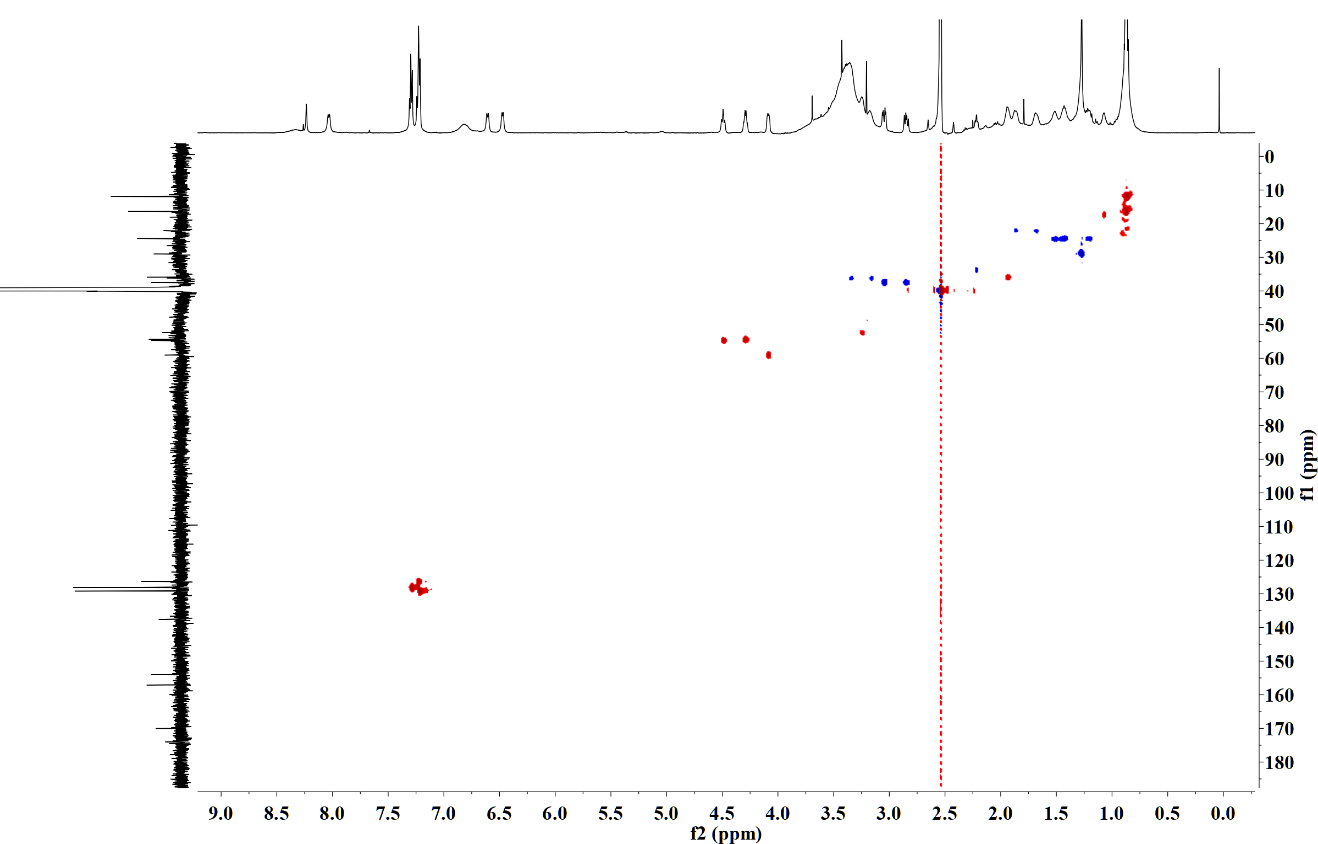


**Figure S13. HSQC spectrum of omicsynin E1 (1).**


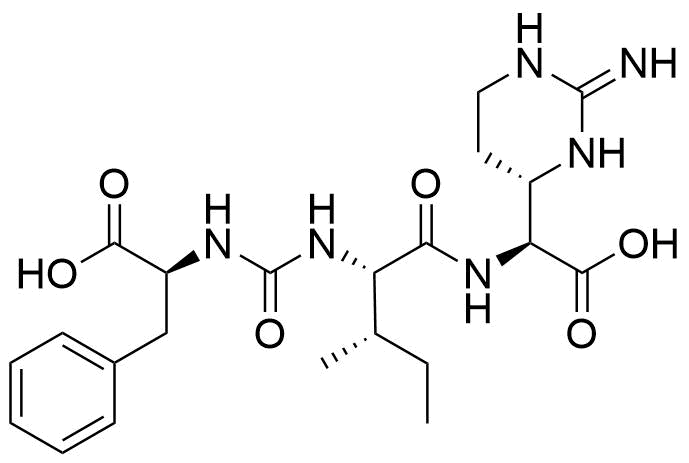

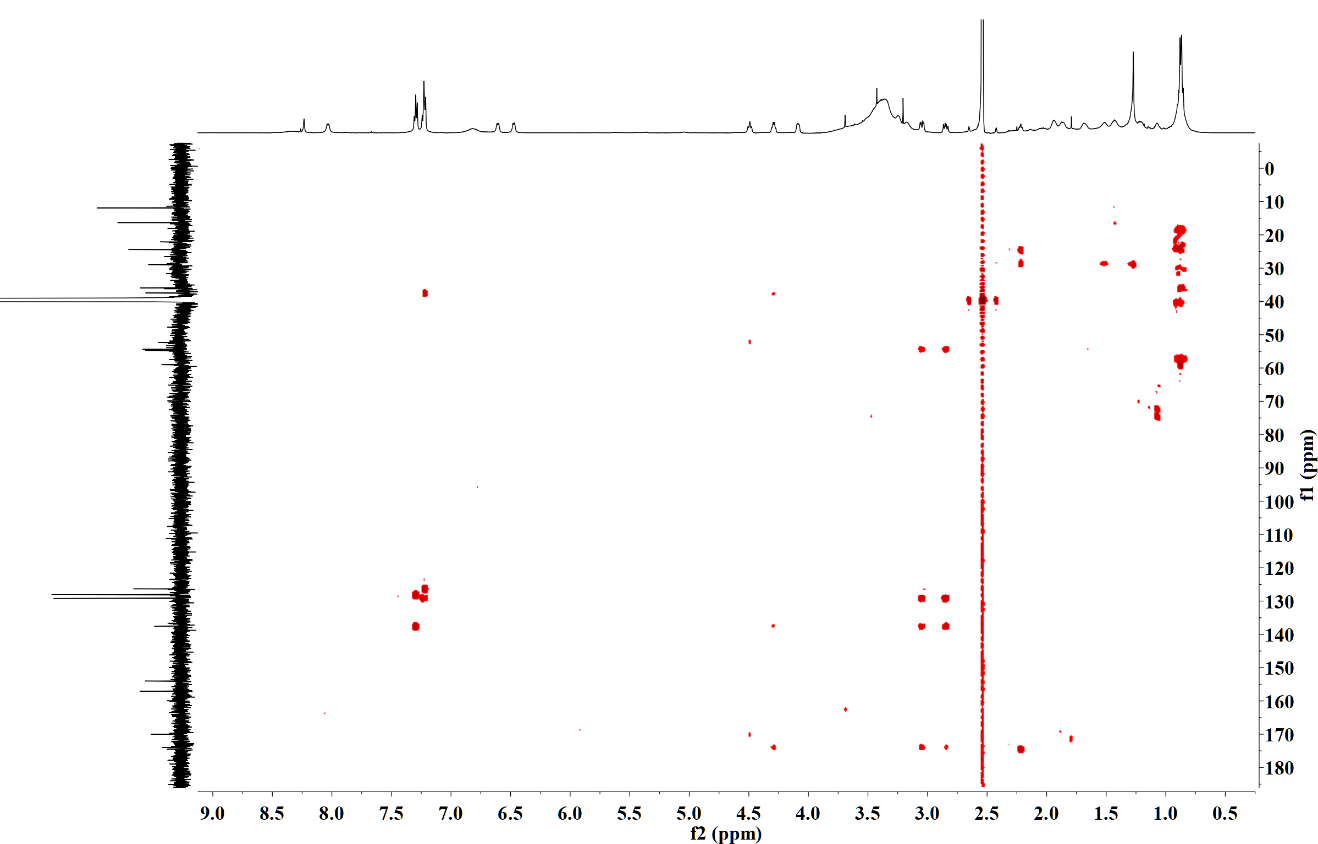


**Figure S14. HMBC spectrum of omicsynin E1 (1).**

**
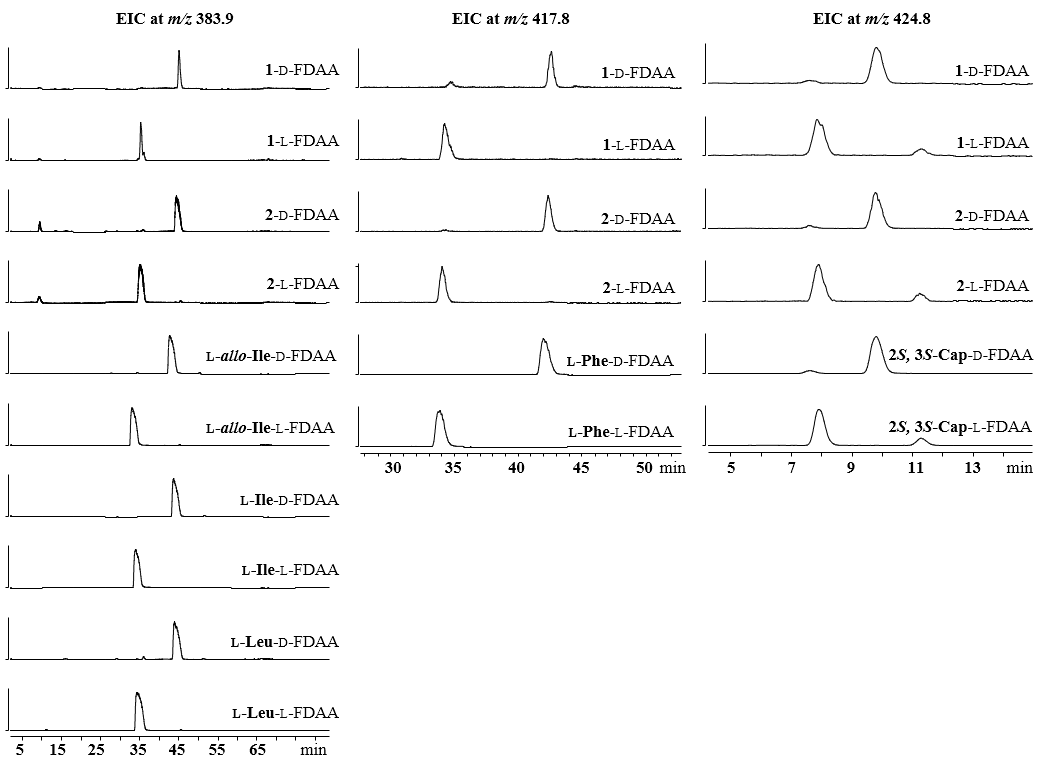
**

**Figure S15. Marfey’s analysis of omicsynin E1 (1) and E2 (2)**

**Reference**

1. Sun, H., Li, X., Chen, M., Zhong, M., Li, Y., Wang, K., Du, Y., Zhen, X., Gao, R., Wu, Y., Shi, Y., Yu, L., Che, Y., Li, Y., Jiang, J. D., Hong, B., & Si, S. Multi-omics-guided discovery of omicsynins produced by *Streptomyces* sp. 1647: pseudo-tetrapeptides active against influenza A viruses and coronavirus HCoV-229E, *Engineering.* 2022; 16: 176–86. https://doi.org/10.1016/j.eng.2021.05.010
2. Paget, M. S., Chamberlin, L., Atrih, A., Foster, S. J., & Buttner, M. J. Evidence that the extracytoplasmic function sigma factor sigmaE is required for normal cell wall structure in *Streptomyces coelicolor* A3(2), *J Bacteriol.* 1999; 181: 204–11. https://doi.org/10.1128/JB.181.1.204-211.1999
3. Bierman, M., Logan, R., O'Brien, K., Seno, E. T., Rao, R. N., & Schoner, B. E. Plasmid cloning vectors for the conjugal transfer of DNA from *Escherichia coli* to *Streptomyces* spp, *Gene.* 1992; 116: 43–9. https://doi.org/10.1016/0378-1119(92)90627-2
4. Hong, B., Phornphisutthimas, S., Tilley, E., Baumberg, S., & McDowall, K. J. Streptomycin production by *Streptomyces griseus* can be modulated by a mechanism not associated with change in the *adpA* component of the A-factor cascade, *Biotechnol Lett.* 2007; 29: 57–64. https://doi.org/10.1007/s10529-006-9216-2
